# Experimental evolution at ecological scales allows linking of viral genotypes to specific host strains

**DOI:** 10.1093/ismejo/wrae208

**Published:** 2024-11-23

**Authors:** María Dolores Ramos-Barbero, Borja Aldeguer-Riquelme, Tomeu Viver, Judith Villamor, Miryam Carrillo-Bautista, Cristina López-Pascual, Konstantinos T Konstantinidis, Manuel Martínez-García, Fernando Santos, Ramon Rossello-Mora, Josefa Antón

**Affiliations:** Department of Physiology, Genetics and Microbiology, University of Alicante, Alicante 03690, Spain; Departament de Genètica, Microbiologia i Estadística, Universitat de Barcelona, Diagonal 643. Annex. Floor 0, Barcelona E-08028, Spain; Department of Physiology, Genetics and Microbiology, University of Alicante, Alicante 03690, Spain; School of Civil and Environmental Engineering, Georgia Institute of Technology, Atlanta 30332, GA, United States; Marine Microbiology Group, Department of Animal and Microbial Biodiversity, Mediterranean Institute for Advanced Studies (IMEDEA, UIB-CSIC), Esporles 07190, Spain; Department of Physiology, Genetics and Microbiology, University of Alicante, Alicante 03690, Spain; Department of Physiology, Genetics and Microbiology, University of Alicante, Alicante 03690, Spain; Department of Physiology, Genetics and Microbiology, University of Alicante, Alicante 03690, Spain; School of Civil and Environmental Engineering, Georgia Institute of Technology, Atlanta 30332, GA, United States; Department of Physiology, Genetics and Microbiology, University of Alicante, Alicante 03690, Spain; Department of Physiology, Genetics and Microbiology, University of Alicante, Alicante 03690, Spain; Marine Microbiology Group, Department of Animal and Microbial Biodiversity, Mediterranean Institute for Advanced Studies (IMEDEA, UIB-CSIC), Esporles 07190, Spain; Department of Physiology, Genetics and Microbiology, University of Alicante, Alicante 03690, Spain; Multidisciplinary Institute of Environmental Studies Ramon Margalef, Alicante 03690, Spain

**Keywords:** virus-host dynamics, hypersaline system, Salinibacter, halovirus, viral evolution

## Abstract

Viruses shape microbial community structure and activity through the control of population diversity and cell abundances. Identifying and monitoring the dynamics of specific virus-host pairs in nature is hampered by the limitations of culture-independent approaches such as metagenomics, which do not always provide strain-level resolution, and culture-based analyses, which eliminate the ecological background and *in-situ* interactions. Here, we have explored the interaction of a specific “autochthonous” host strain and its viruses within a natural community. Bacterium *Salinibacter ruber* strain M8 was spiked into its environment of isolation, a crystallizer pond from a coastal saltern, and the viral and cellular communities were monitored for one month using culture, metagenomics, and microscopy. Metagenome sequencing indicated that the M8 abundance decreased sharply after being added to the pond, likely due to forces other than viral predation. However, the presence of M8 selected for two species of a new viral genus, *Phoenicisalinivirus*, for which 120 strains were isolated. During this experiment, an assemblage of closely related viral genomic variants was replaced by a single population with the ability to infect M8, a scenario which was compatible with the selection of a genomic variant from the rare biosphere. Further analysis implicated a viral genomic region putatively coding for a tail fiber protein to be responsible for M8 specificity. Our results indicate that low abundance viral genotypes provide a viral seed bank that allows for a highly specialized virus-host response within a complex ecological background.

## Introduction

Virus-host interactions shape microbial ecosystems [1], have profound effects on food webs and biogeochemical cycles, and are key determinants of microbial evolution thus linking evolutionary and ecological dynamics in microbial communities. Virus-host interactions are frequently very specific, both for the host and the virus, and depend on the community context [2]. At the level of individual virus-host pairs, interactions may proceed in a continuum between two extreme infection strategies: purely lytic and purely lysogenic [3, 4]. These interactions may translate, respectively, into two large scenarios in nature, known as Kill the Winner (KtW) and Piggy back the Winner (PbtW). In the KtW model, lytic infections are favored when host availability increases, keeping host diversity constant [5], whereas in the PbtW this increase will favor lysogeny and its ecological consequences. Both models have experimental support in different conditions and settings [6–8]. However, these are likely oversimplifications of the reality in which mixed approaches are adopted, either consecutively or simultaneous in different components of the community [9, 10], in combination with less characterized viral strategies such as pseudolysogeny or chronic infection [3]. Most importantly, how these interactions proceed have a strong dependence on the ecological communities involved and their biotic and abiotic interactions [1]. Compatible with these two scenarios, the Bank model suggests that most viral variants are present in the natural community at very low abundances and they only become (transiently) abundant when susceptible host increase their availability [11, 12].

The specificity of virus-host interactions may be undetectable using some community-focused approaches. Powerful as it is, short-read metagenomics cannot always assemble genomes from abundant viruses if levels of microdiversity are high. In fact, one of the most notorious cases is that of 37F-6, a marine virus that putatively infects *Pelagibacter* and is likely one of the most abundant viruses on the planet. This virus was unveiled only by the use of single-virus genomics since its genome had never been assembled from marine viral metagenomes due to its high level of microdiversity [13, 14]. Likewise, pairwise culture analyses, which have unparalleled power to explore interaction mechanisms, eliminate the community within which the virus-host interactions occur [1]. The temporal monitoring of medium complexity systems, such as mesocosms, can overcome some of the limitations of metagenomics and cultivation-based studies. This mixed approach allows the study of relatively controlled and natural systems and provides a sort of controlled “natural” experimental evolution setting [15].

Previously, some studies have been carried out to explore the evolutionary effects of the (normally simplified) community presence on focal cultured bacteria-phage species [2]. However, as pointed out by Ignacio-Espinoza et al. [16], “direct field observations of co-evolutionary outcomes are rare or limited to model systems”. Here, we have explored the interaction of a specific “autochthonous” host strain within a natural and diverse virus community, providing insight on the eco-evolutionary feedbacks occurring during virus-host dynamics. The system under study has been hypersaline brines (close to saturation) of a crystallizer pond from Es Trenc solar salterns in the Mallorca island (Balearic Islands, Western Mediterranean). The extreme environmental conditions in hypersaline ecosystems appear to drive their microbial communities into taxonomically similar assemblages with low diversities that are accompanied by highly dense viral communities. These prokaryotic assemblages generally consist of two major lineages: the archaeal *Halobacteria* class and the bacterial family *Salinibacteraceae* [17, 18]. Thus, hypersaline environments constitute tractable systems for studying virus-host interactions in natural settings. Within the *Salinibacteraceae* family, *Salinibacter ruber* was the first member of *Bacteria* demonstrated to grow actively and to high abundance in brines [19]. It is the dominant bacterium in many hypersaline systems worldwide although generally it is outnumbered by haloarchaea, in spite of being as halophilic as the most halophilic archaeon. This lower abundance could be due to viruses that control *Sal. ruber* populations in nature [20, 21].

Within the above mentioned framework, our goal was to explore the effects of the re-introduction of an autochthonous host on its natural community of cells and viruses. For this purpose, we added *Sal. ruber* strain M8 to a crystallizer pond from Es Trenc saltern, from where the strain had been isolated 15 years before, and monitored the community for one month. Our results showed how the increase of a suitable host was followed by a selection of initially very low abundance viral strains encoding specific genomic islands putatively involved in host recognition. In addition, drastically different outcomes were achieved by metagenomics and culture even for culturable host strains, stressing the complementarity of both approaches. The novelty of our work lies on the temporal monitoring of the evolution of phage-host interactions at the strain level against an ecological background, away from the lab environment.

## Materials and methods

### Experimental setup (pond description and sampling)

**Figure 1:**
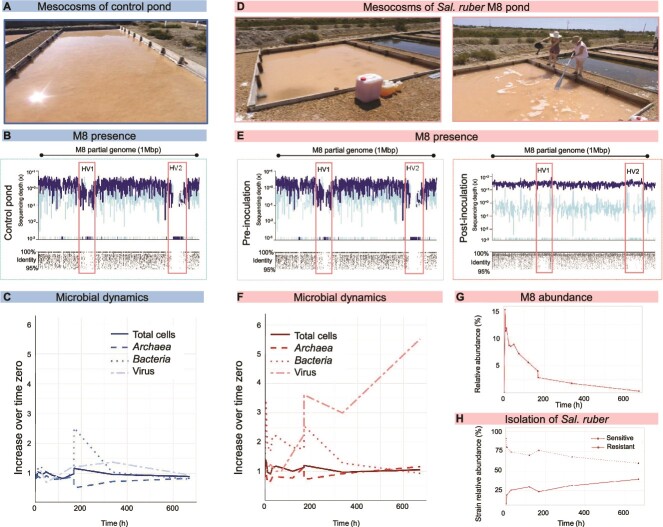
**The experiment**. (A) Control pond. (B) Recruitment of the genomic region of *Sal. ruber* containing the strain specific regions HV1 and HV2 against reads from the metagenome retrieved from the control pond at the beginning of the experiment. (C) Microbial dynamics in the control pond throughout the experiment tracked using total cell counts by DAPI staining, archaea and bacteria counts by CARD-FISH with domain specific probes, and virus counts as VLP detection by Sybr gold staining followed by epifluorescence microscopy. For each parameter, the variation respect to the initial time is given. (D) Pond amended with the *Sal. ruber* M8 culture. (E) Recruitment of the genomic region of *Sal. ruber* containing the strain specific regions HV1 and HV2 against reads from the metagenomes retrieved from the M8 pond before (t = 0) and after (t = 4 h) adding the strain M8 to the pond. (F) Microbial dynamics in the M8 pond measured as shown for the control pond in B. (G) Dynamics of M8 in the pond throughout the experiment measured as percentage of metagenomic reads recruited by the strain specific regions. (H) *Sal. ruber* strains isolated from the M8 pond which were sensitive or resistant to *Phoenicisalinivirus*.

The mesocosm experiment was performed in August 2014 at the Mediterranean solar salterns of Es Trenc, located in Mallorca Island, Spain (39°20’N; 2°59′E), where two crystallizer ponds like the ones used for salt exploitation were prepared (Figs. 1A and 1D). The first crystallizer, called Control pond, was used to study the natural dynamics of microbial communities and was left unamended along the experiment. The second one, named *Sal. ruber* strain M8 pond, was inoculated with 1.83X10^13^ cells of *Sal. ruber* M8 pure culture, ~9.3% of total cell counts (see below for the inoculum preparation details). The volume of brine in each ponds was, approximately, 15.2 m^3^ for the control pond, and 7.2 m^3^ for the amended pond. Both ponds were filled up to 40 cm approx. The ponds were built in 2012 and have been operating as the rest of crystallizers in the salterns since then, using the brines from the system of concentrator ponds that had been working for decades. *Sal. ruber* M8 was isolated from the Es Trenc saltern 15 years before the experiment [19] but was below the detection limit at the time of inoculation as checked using strain specific primers 156 [22].

The experiment was monitored for one month and the ponds were periodically sampled (including the sampling time right before and after *Sal. ruber* M8 addition, see Supplementary Fig. S1). During the first week, samples were taken every 24 h and then weekly. A total of 13 brine samples were taken from each pond, five of which were studied in more detail. At 168 h, because they were drying out due to evaporation, ponds were refilled (using the same brine for both ponds), a standard procedure in solar saltern management.

Biological replicates were not obtained due to the large volume and frequent sampling required in the mesocosms, making replication prohibitively expensive. Moreover, replication is generally unnecessary in time-series experiments like ours, where microbial community variability tends to be relatively small [23], as shown in previous time-series studies of hypersaline samples [24].

### *Sal. ruber* M8 inoculum preparation and isolation along the experiment

A pure culture of *Sal. ruber* M8 was grown in 25% sea water (SW), 0.2% yeast extract (YE), pH 7.2 at 37°C as previously published [19]. The inoculum was checked using strain-specific primers [22] and a pool of primers specific to *Salinibacter* spp. or Archaea to guarantee its purity. Finally, 64 liters of pure M8 inoculum were concentrated to 35 liters by Vivaflow 100 K ultrafiltration in order to facilitate its transport from Alicante to Mallorca. Inoculum cell concentration was determined by DAPI staining [25].

*Sal. ruber* M8 was isolated along the experiment by plating brine samples in 25% SW + 0.05% YE agar plates at 37°C and checking the individual colonies with M8 specific primers.

### Isolation of M8 viruses from mesocosm experiments

Exponentially grown M8 cultures were directly mixed with 0.7% agar in 25% SW and poured into 25% SW + 0.2% YE agar plates. Viral assemblages were prepared after centrifuging brines to pellet cells at 13000 rpm and filtering the supernatants through 0.22 μm filters. Then, 5 μl of these viral preparations, were separately spotted, directly and after decimal serial dilution (down to 10^−6^ of the initial concentration), on top of the *Sal. ruber* M8-soft agar layer. Virus isolation was carried out with samples from M8 and control ponds, as well as from another crystallizer pond of the system (E2), amended with *Sal. altiplanensis* strain PH3 [26], which was not otherwise used in this work. The resulting spot-plaques were then collected from the soft agar, suspended in 25% SW, mixed thoroughly, and used for further virus purification using the double layer technique (See Supplementary Methods for more details of the process).

### Microbial and viral counts and nucleic acid extraction

Cells and viruses were counted by DAPI and Sybr Gold staining [20, 21, 27], and the proportion of Archaea and Bacteria determined by FISH (Antón et al., 1999). Details are provided in the supplementary methods.

For cell DNA extraction, 25 ml of brine samples were centrifuged at 13000 rpm and DNA was extracted from the pellet as detailed previously (Urdiain et al., 2008). For RNA extraction, pellets were stored in RNAlater solution (Invitrogen, Thermo Fisher Scientific, Lithuania) at −80°C until processed. The PowerMax Soil DNA kit for extracting RNA was used for total RNA extractions (MoBio) and TURBO DNAse (Ambion) to remove DNA. More details are provided in the supplementary methods.

### Sequencing and preliminary viral metagenome analyses

Sequencing of viral DNAs was performed using a MiSeq Nextera XT run (2x300bp, paired-end reads; Illumina) and isolated viral genomes were sequenced separately using Nextera libraries and a MiSeq SEQ 500 Hi-output sequencing run (Illumina). Reads were quality assessed and trimmed using PRINSEQ v0.20.4 software [28] (min_length 50, trim_qual_right 20, trim_qual_type mean, trima_qual_window 20) and paired-end reads were joined using fq2fa from IDBA v1.1.1 assembler [29]. Only reads larger than 50 bp and with a quality over 20 were considered for further analyses. The Nonpareil v2.4 tool [30] was used to estimate the coverage of the community in each metagenome dataset with default parameters. De novo assemblies of trimmed reads were generated using the IDBA v1.1.1 assembler [29] with the “-pre_correction” option.

### Sequencing and initial cell metagenome and metranscriptome analyses

Sequencing of cell DNA was performed using an MiSeq (2 × 250 bp, paired end reads; Illumina) and HiSeq (2 × 100 bp, paired end reads; Illumina) instruments. SolexaQA tool v3.1.4 [31] was used to trim the paired-end reads with a quality score below 20 and reads with <50 bp lengths were discarded. The trimmed reads were assembled with the IDBA v1.1.1 assembler [29] with the “—pre-correction” option. Genes from the assembled contigs with length > 500 bp were predicted using MetaGeneMark.hmm v3.25 [32]. Protein-coding genes were annotated against the UniProt database using BLASTp 2.2.30+ [33], considering the best match to have >40% amino-acid identity, >70% sequence length compared to the reference sequence and a bit-score higher than 60. For phylogenetic purposes, the reads associated with 16S rRNA genes were extracted using the Parallel-META v2.4 tool [34] and clustered at 98.7% similarity using QIIME v1.9.1 script *pick_closed_reference_otus.py* to identify Operational Taxonomic Units (OTUs). The representative sequences from each OTU were aligned using the SINA tool v1.3.1 [35] and were added to the reference database SILVA REF 128 [36] using the parsimony method implemented in the ARB software v6.0.6 [37]. The OTUs were then clustered into OPUs (Operational Phylogenetic Units) as recommended [38].

For metatranscriptome analyses, RNA was retrotranscribed to cDNA and sequenced. Metatranscriptomic sequences were cleaned by Prinseq v0.20.4 (−fastq -fastq2 -verbose -derep 12 345 -lc_method entropy -lc_threshold 70 -out_format 3 -min_len 20 -trim_qual_right 15 -trim_qual_left 15 -trim_qual_window 2 -trim_qual_step 1 -min_qual_mean 20 -ns_max_p 1 -noniupac -out) (Cantu et al., 2019). Following, the 16S rRNA gene sequences were identified and removed by rnascan v 2.0.0 (−moltype ssu,lsu,tsu). The trimmed metatranscriptomic reads were mapped against *Sal. ruber* M8 and M8 isolate virus genomes by Bowtie v9.3.0 (Langmead and Salzberg, 2012) and Tophat library-type fr-firststrand —no-novel-juncs (Trapnell et al., 2009). The outputs were filtered using the Samtools v1.10 collection (depth –aa –d 10 000 000) (Weeks and Luecke, 2017).

### Cell and virus metagenome annotation and comparison

Functional annotation of predicted genes from cellular and viral assembled metagenomes was done using the JGI IMG/MER platform [39], DIAMOND BLASTp v0.9.28. [40] and interpro 5.41–78.0 [41]. In addition, the NR NCBI database (December 2019 update) and the UniProt database [42] were used for viral and cell metagenomes, respectively. Raw cell and viral metagenomes “all versus all” comparisons were performed by BLASTn 2.9.0 stand-alone considering only best-hit matches over 70% of read coverage. Additionally, metagenome raw read comparisons were performed by MASH v1.1 (parameters for sketch: -s 10000 -r -m 2) [43] and Metafast v1.3 [44].

### Viral genome comparisons and clustering

After viral DNA extraction and sequencing (see supplementary methods.), whole viral genome comparisons (in nucleotides) were performed by VIRIDIC [45] and ORF comparisons between reference viral genomes were analyzed using an amino acid reciprocal best match strategy (rbm.rb, enveomics collection) [46]. Heat maps and boxplots were drawn by Plotly by R (Plotly Collaborative Data Science 2015 Data Storytelling Studio @ MIT). Whole viral genome alignments were drawn using the Easyfig platform [47]. Viral proteomic tree including published viral genomes was built using VIPtree [48].

The first clustering step was performed using cd-hit-est v4.8.1 (−c 1, n 8, aL 0.8) [49], considering only genomes larger than 43 kb (this threshold was chosen based on the viral genome sizes determined by PFGE). Briefly, ANI was performed by VIRIDIC [45] and Jspecies [50]. The non-redundant 120 viral genomes were clustered again according to their reciprocal AAI (average amino acid identity). The AAI of each genome was calculated by reciprocal best match of all versus all ORFs in amino acids (rbm.rb, default parameters by enveomics collection [46] and additionally the ANI of each complete genome was calculated. Finally, viral genomes were clustered using a dissimilarity matrix based on AAI values between genome pairs (Bray Curtis) [51] and the largest and most complete genome of each cluster was selected as “group reference genome”.

### Cell and viral genome abundances

Cell and viral abundances, as well as the absence, presence, and permanence of M8 during the experiment were calculated by fragment recruitment of each genome in each metagenome (cell or virus) by BLASTN v2.9.0 (cut-off >70% coverage, −evalue 0.1 and filtered with the BlastTab.best_hit_sorted.pl script from the enveomics collection). The BlastTab.seqdepth.pl script, also from the enveomics package [46], was used to calculate the sequencing depth of the genomes. Isolated viral abundances (viral genotype abundance) were calculated as a percentage of total recruited nucleotides in each metagenome (% of total nucleotides were performed as recruited nucleotides/total metagenome nucleotide size × 100) and normalized by viral genome size (Kbp), only reads mapped over 70% of coverage and 100% of identity were considered. Recruitment plots were drawn with R (enveomics.R library) [46]. Finally, the abundance fold change between different times was calculated as (abundance in time X/ abundance time Y) when X is a selected time point n and Y is the previous time n-1.

### Proteomic analyses of the new viral isolates

In order to refine the annotation of the viral genomes, structural proteins (i.e. present in the virion) were analyzed by orbital mass spectrometry on digested virion extracts. The detected proteomes were mapped into a custom proteomic database including the non-redundant ORFs from isolated viral genomes. More details are provided in the supplementary methods.

### Viral genome diversity analyses (SNPs and pN/pN + pS)

To calculate the percentage of polymorphisms in the isolated viral genomes, raw reads from each viral metagenome were mapped against isolated viral genomes separately (only reference genomes) using the sensitive local mode of Bowtie2 V 2.4.4 (Langmead and Salzberg, 2012). Then, in order to obtain counts of synonymous and nonsynonymous mutations in each ORF, the output files were analyzed by DiversiTools (−-min_aa_cov 5 --min_mut_codon_count 4 --min_mut_codon_freq 1) (http://josephhughes.github.io/DiversiTools/). Only ORFs shared between the control and M8 ponds were considered. Details on downstream analyses are provided in the supplementary methods.

### qPCR and amplicon sequencing analyses of selected viral genes

The abundance of the newly isolated *Phoenicisalinivirus* was measured in the control and M8 pond by qPCR with two primers pairs targeting, respectively, the highly conserved tail fiber protein gene (primers C) and a highly diverse structural protein gene (primers VR, from “variable region”). All qPCR amplifications, including those of the standard curve, were carried out in triplicate. See supplemental methods for further details.

The diversity of these viruses was monitored by a metabarcoding analysis using these two primers pairs with the appropriate adapter for Illumina sequencing. More details are provided in the supplementary methods. Sequences were quality filtered by employing Trimmomatic v0.36 [52] (ILLUMINACLIP:NexteraPE-PE.fa:2:30:10 LEADING:3 TRAILING:3 SLIDINGWINDOW:4:15 MINLEN:36) and joined with FLASH v1.2.11 using default parameters [53]. To remove spurious sequences, only those sequences that aligned against viral genomes (BLASTn v2.9.0+, minimum e-value 1e-10) were analyzed. FastA.sample.rb from the Enveomics collection [46] was employed to normalize samples to 11 421 sequences for primers C, and 61 195 sequences for RV to allow comparisons between them. Then, sequences were clustered at 100% identity and coverage with cd-hit-est v4.8.1 (−as 1 -c 1) [49], the Shannon index was calculated by the phyloseq R package v1.40.0 [54] and finally, the plot was drawn in R with ggplot2 v3.4.2 [55].

### Host range

The host ranges of the 10 reference viruses against a panel of different *Sal. ruber* strains were determined by the spot test technique [21]. The susceptible and resistant hosts were detected with a spot test applying cell-free phage lysates over agar-overlays of individual host lawns. Susceptibility to the viruses was verified by plaque assay. The plates were incubated as described above.

## Results and discussion

### Experiment set-up and monitoring community dynamics

Two ponds (hereafter “control” and “M8 pond”, Figs. 1A and 1D) were chosen for the experiment based on the undetectable levels of strain *Sal. ruber* M8, as determined by PCR with M8-specific primers, which can detect in the order of 10^3^ cells/ml (as estimated by using serial dilutions of a *Sal. ruber* M8 culture), and further confirmed by metagenomic analyses (Figs. 1B and 1E 1, see below). M8 cells from a pure culture were added to the M8 pond to a final concentration of 9.3% of total cells whereas nothing was added to the control pond. Strain M8 had been isolated in 1999 from these salterns [19], and therefore can be considered part of their autochthonous microbiota and had the opportunity to interact and coevolve with the salterns’ cell and virus assemblages in the past.

The ponds were monitored during a 672-h period, with a higher sampling frequency at the beginning of the experiment (Supplementary Fig. S1). At 168 h, due to evaporation, the ponds were drying out and had to be refilled (using the same brine for both ponds, which caused a decrease in salinity of 4–7% units approx., see Table S1), a standard procedure in solar saltern management. It is important to notice that the refilling brine came from the concentrator pond used for feeding all the crystallizers. In both M8 and control ponds, cell counts returned to their time zero numbers by the end of the experiment while virus counts increased only in the M8 pond (Figs. 1C and 1F, and Table S1).

The fate of M8 in the M8 pond was followed by metagenomic recruitment of M8-specific genomic regions, known as hypervariable regions 1 and 2 [22], labeled as HV 1 and 2 (Figs. 1B and 1E, and S2). This analysis also confirmed the absence (or abundance below the sequencing detection limit, that at this sequencing depth is 2.10^5^ genomes/ml calculated as in [56]) of the strain in the system prior to the experiment (Fig. 1E, Fig. S2A), in good agreement with the previous PCR with strain specific primers. Following M8’s addition to the pond, its abundance steadily decreased, reaching, by the end of the experiment, an almost residual abundance (less than 0.4% of the metagenomics reads). This decrease of M8 did not imply that the added M8 was inactive, as shown by genome recruitment against metatranscriptomic reads of the cellular assemblage (Fig. S2B). The decrease of M8 density is predicted by classical ecological theory [57] and can be explained as an outcome of either competition and/or viral predation.

The analysis of cellular and viral metagenomes (Tables 1 and 2 for their general traits) indicated how the addition of M8 impacted both cellular and viral assemblages (Fig. S3); distances between whole metagenome read comparisons (MASH distances in Figs. S3A, B, and D) showed that M8 addition had a larger effect on the cellular than the viral community (Fig. S4). However, although the initial communities were different in each pond, pointing to a certain degree of stochasticity in the system, the taxonomic profiles of both crystallizers at the end of the experiment were very similar to that of their respective initial communities (Fig. S3C). A similar stability was observed in different experiments carried out in 2012 and 2014 in crystallizer ponds from the saltern system analyzed here [24].

**Table 1:** Characteristics of the viral metagenomes used in this study.

**Pond**	**Metavirome ID**	**Time (hours)**	**Reads**	**Total nucleotides**	**%GC**	**Nonpareil values (diversity/coverage)**	**16S rRNA gene proportion (%)**	**Assembly size (Mbp)**	**Q20/Q30**	**Min/max (bp)**	**Contigs**	**Assembled (%)**
Control pond	Control T0h	0	1 314 648	271 550 874	53.5	16.24/87.87%	0.018	26.5	98.4/93.4	100/22350	62 040	21.5
	Control T72h	72	1 307 290	262 782 463	55.5	16.9/76.7%	0.05	26.7	98.5/93.6	100/40208	595 588	21.4
	Control T168h	168	1 340 470	262 122 041	55	16.78/83.4%	0.04	25.7	98.6/94	100/28633	54 615	21.3
	Control 169 h	169	1 498 842	304 765 505	54.4	16.52/86.1%	0.05	29.7	98.5/93.6	100/34056	60 010	24.5
	Control T672h	672	731 830	145 710 123	57	16.5/82.1%	0.05	15.49	98.5/93.7	100/23854	31 382	20.9
*Sal. ruber* M8 pond	*S. ruber* M8 T0h	0	990 508	220 721 760	53.7	16.9/90.4%	0.002	21.0	98.3/96	100/18155	57 625	24.1
	*S. ruber* M8 T72h	72	2 611 956	575 669 555	54.8	15.7/93.6%	0.005	4.8	98/91	100/31905	149 598	24.7
	*S. ruber* M8 T168h	168	1 961 392	439 592 060	50.3	17.1/93.3%	0.001	36.42	98/91.8	100/38302	111 380	24.7
	*S. ruber* M8 T169h	169	1 859 328	407 296 917	54.4	16.6/88.2%	0.001	40.5	98.4/93	100/34260	100 265	25.5
	*S. ruber* M8 672h	672	1 977 784	438 021 570	52.2	16.5/92.1%	0.001	45.2	98.5/93.4	100/263015	108 222	28.9

**Table 2:** Characteristics of cellular metagenomes.

**Pond**	**Metagenome ID**	**Time (hours)**	**Reads**	**Total nucleotides**	**%GC**	**Nonpareil values (diversity/coverage)**	**Assembly size (Mbp)**	**Contigs**
Control pond	Control T0h	0	337 702	66 195 891	55.738	17.77/42.1%	6.3	4555
	Control T72h	72	528 468	103 434 888	56.441	18.08/44.61%	10	6885
	Control 169 h	169	997 812	196 400 422	55.217	17.77/58.23%	17	11 691
	Control 360 h	360	788 212	151 486 662	56.668	18.18/48.89%	13	10 898
	Control T672h	672	1 142 478	229 346 011	55.963	18.11/55.66%	19	14 875
*Sal. ruber* M8 pond	*S. ruber* M8 T0h	0	497 798	95 137 015	57.082	18.1/43.86%3	12	9248
	*S. ruber* M8 T72h	72	453 732	95 392 451	58.540	17.61/52.62%	12	7539
	*S. ruber* M8 T168h	168	384 504	83 688 457	57.584	17.91/44.22%	11	7909
	*S. ruber* M8 T169h	169	40 273 294	3 797 761 300	61.898	18.2/85.95%	94	64 502
	*S. ruber M8 t 360 h*	360	28 844 742	2 708 447 940	58.114	17.97/87.4%	71	51 579
	*S. ruber* M8 672h	672	110 992 232	2 209 258 135	55.4	18.08/83.5%	94	85 892

### Retrieval of viruses infecting *Sal. ruber* M8

Two strategies were used for M8 virus retrieval: the identification of metagenomic viral contigs corresponding to putative *Sal. ruber* viruses and the isolation of *Sal. ruber* M8 viruses by plaque assays.

Viral contigs were retrieved from the viral metagenomes of both the control and the M8 ponds (Dataset S1, Fig. S5), and their relative abundances along the experiment were calculated. Only contigs larger than 10 Kb whose relative abundances increased along the experiment more in the M8 than in the control pond were selected for further study. The rationale was to select genomes corresponding to virulent viruses that would increase their abundance as a result of M8 infection and lysis. However, except for their relatively high GC content (also common in most haloarchaeal viruses), genomic analyses did not unveil any trait (e.g. CRISPR protospacers and tRNAs) that could unambiguously identify them as infecting *Sal. ruber*. Therefore, this strategy was disregarded, and a culture-dependent approach was undertaken instead.

Viruses infecting M8 were isolated by the plaque assay technique using filtered brine as a direct source of viruses, as previously described ([21]; methods). Viruses infecting M8 could be isolated from the M8 pond, at time points 336 (40 plaques) and 672 (132 plaques). Not a single M8 virus was isolated from control pond brines (Dataset S2 and Table S2). Furthermore, an additional set of 27 viruses infecting M8 could be isolated from a contiguous pond (named E2, supplementary results), after 672 h post inoculation. Thus, a total of 199 plaques were obtained from three different samples (Dataset S2).

### Genomic characterization of the *Phoenicisalinivirus*: A new genus of viruses infecting *Sal. ruber* M8

A total of 190 virus genomes were sequenced and, of these, 120 were non-redundant complete viral genomes (Table S2). Overall, the 120 genomes formed a genetically homogenous group, which, according to VIRIDIC (Fig. S6A), constituted two species from the same viral genus. One species was represented by only one genome, B2_17, whereas the second species comprised the other 119 viruses, which formed nine different groups (i.e. intra-species clusters) based on amino acid identity (Fig. S6B). One representative of each of the nine groups (i.e. nine strains from the same species), together with virus B2_17, were selected for detailed phenotypic and genotypic characterization. Virions from each representative had a head and tail morphology resembling myoviruses (Fig. S7) with head diameters around 66 nm and tail lengths of 120 nm, slightly larger than the previously isolated *Sal. ruber* phages [21]. Viral particles were not sensitive to chloroform (supplementary methods for the protocol description), likely indicating that they had no lipid envelopes.

**Figure 2:**
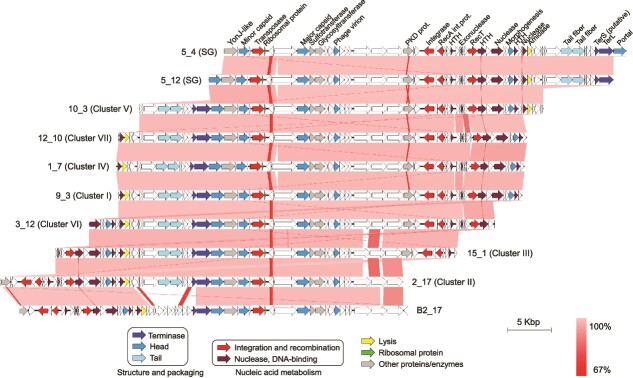
**The *Phoenicisaliniviruses*.** Genomic organization of the phoenicisaliniviruses. Every ORF is colored according to its predicted function (see legend). Lines connecting genomic regions are colored according to their nucleotide identities.

The genomes of the newly isolated viruses infecting *Sal. ruber* M8 had sizes and GC contents of 44–46 Kb (confirmed by PFGE) and 63%, respectively, and presented terminal redundancy. They had no homology at the nucleotide level with any previously isolated virus present in databases, including those infecting *Salinibacter* (Fig. S8 for a proteomic tree). According to ICTV guidelines (two phages are assigned to the same species/genus if their genomes are more than 95%/70% identical at the nucleotide level over their full genome length, tested reciprocally [58–60]), these viruses belonged to two new species (Fig. S8) within the *Caudoviricetes* class, and formed a new genus for which we propose the name ***Phoenicisalinivirus**.* The genus name comes from the Phoenix bird in the Greek mythology which regenerated from its ashes cyclically. The two new species are named *P. gymnesicum* and *P. balearicum*, in reference to the Greek and Latin names, respectively, of the place of isolation (Mallorca, Balearic Islands, Spain). Around one half of the ORFs in the new *Phoenicisalinivirus* genomes could be annotated, either *in silico* or by the identification of structural proteins by mass spectrometry (Fig. 2, Supplementary Dataset S3). A more detailed description of the genomic characteristics of *Phoenicisalinivirus* is provided in Supplementary Results.

The genomes of both viral species displayed regions of high gene identity and synteny, as well as species-specific regions corresponding with the metagenomic islands discussed below, as an additional example of the typical mosaicism observed in viral genomes (Fig. 2). The comparison of the representative viral genomes within the species *P. gymnesicum*, from which we had a high number of genomes to compare (i.e. 119 genomes in the nine clusters mentioned above, whereas only one genome corresponded to *P. balearicum*), indicated that, despite their relatedness, there were genomic regions where differences accumulated whereas other regions were extremely conserved (Fig. 2 and Supplementary Fig. S9). Genes coding for proteins identified as structural were overall more conserved than genes encoding other or unknown functions (Fig. S9, the equivalence in genes labeling among the different isolates can be found in Dataset S3). Conversely, several hypothetical proteins displayed high levels of variation between the different virus groups. A similar trend has been observed when studying uncultured viruses infecting a natural bloom of green sulfur bacteria [6]. These regions of variability do not seem to affect host specificity of *P. gymnesicum* although they could potentially affect future adaptation to new hosts, as shown by experimental genomic evolution of phage-host pairs [61].

None of these genomes had any similarity to the viral contigs (larger than 3 Kb) assembled from their corresponding viral metagenomes, likely due to the high microdiversity of the population and low relative abundance (e.g., see recruitment plots in Fig. S10), together with the presence of both species-specific and shared genomic regions. As mentioned above, this is a well know limitation of viral metagenomics [21, 62, 63].

To ascertain to which extent these isolated viral genomes represented their “natural” populations and could thus be used for a meaningful monitoring along the experiment, we first retrieved these populations by selecting all metagenomic reads mapping above 95% identity with the isolate genomes in the metavirome of the *Sal. ruber* amended pond at time 692 h. This value has been previously considered as the threshold for a viral species/population [64, 65]. Then, the fraction of the population retrieved by culture was ascertained by quantifying the reads matching with 100% identity against the isolate genomes. The rationale was thus to retrieve first the reads accounting for each viral species-like population and then to ascertain the fraction of these reads corresponding to the isolated genomes. The viral isolates of the species *P. gymnesicum* and *P. balearicum* represented around one half of the diversity (46.9 and 46.4% of the reads representing either species, respectively) in the viral metagenomic reads from their respective natural populations. In other words, culture allowed the retrieval of around one half of the natural species-like population diversity for both viral species. Thus, the isolated viral genomes could be considered as good representatives of their natural populations and were used to monitor viral-host dynamics.

### Dynamics of phoenicisaliniviruses involves the selection of a viral genomic variant

Changes in phoenicisaliniviruses assemblages along the experiment were monitored by recruiting isolated genomes against cellular and viral metagenomic reads from the control and M8 ponds. The recruitment plots showed some genomic regions of low recruitment at the beginning of the experiment that, in pond M8, subsequently experienced an increase in coverage. Thus, complete *P. balearicum* and *P. gymnesicum* genomes could not be detected by metagenomics in the pond before inoculation, although closely related viruses (i.e., sharing part of their genomes with phoenicisaliniviruses) were present. The following analyses focus on the dynamics of the species *P. gymnesicum*, given also that the dynamics of *P. balearicum* was similar (see the corresponding recruitment plots in Fig. S10).

**Figure 3:**
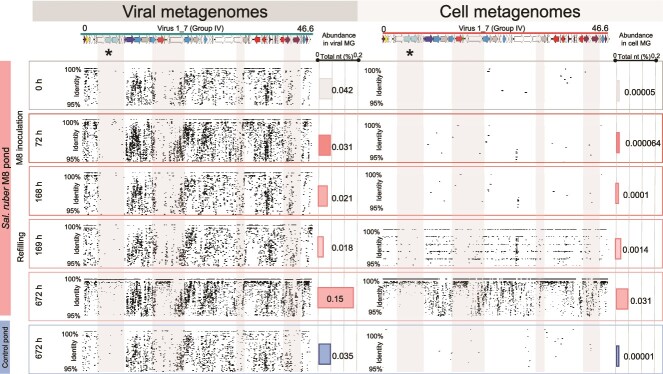
**Dynamics of *Phoenicisalinivirus gymnesicum* throughout the experiment.** Recruitment of reads to a *Phoenicisalinivirus gymnesicum* genome. Reads are from the viral (left) or cellular (right) metagenomes sampled at different times during the experiment in the M8 pond. The asterisk marks the region coding for the putative tail protein.

In more detail, a region specific to the *P. gymnesicum* genome and highly conserved in all isolated viruses (marked with an asterisk in Fig. 3) showed extremely low coverage at the beginning of the experiment in both ponds, and was replaced at the end by a homogenous sequence coverage only in the amended pond (Fig. 3). This was observed for the recruitments with both the viral and the cellular metagenomes from the amended pond, indicating that the selected genomic variants were present both within extracellular virions and actively replicating within the host cells. This pattern was not observed in the control pond (see the corresponding recruitment plots in Figs. 3 and S10).

Annotation indicated that, among the genes within this conserved region, there were two ORFs (colored light blue in Fig. 2) identified as “putative tail fiber protein”. Furthermore, the corresponding proteins could be detected by mass spectrometry in the isolated phages and were therefore structural. Tail fiber proteins are very frequently involved in host recognition and may be responsible for virus specificity [66]. Thus, this genomic region could be involved in host interaction and be responsible for specificity of *P. gymnesicum* for their host. Our results suggest that a genome encoding a variant of the tail fiber protein was selected throughout the experiment, as its sequence became less diverse (as indicated by the recruitment plots) and more abundant over time, as previously shown in other systems [1]. Another clue that suggests this region was involved in host recognition was provided by the genomic comparison of the two isolated virus species which indicated that both viral species differ in this region and had (slightly) different host ranges (Fig. 2), as discussed below. Thus, metagenomic recruitments of the isolated viruses suggested that an assemblage of closely related genomic variants of *P. gymnesicum* with the ability to infect M8 was selected along the experiment.

### Tail fiber gene amplicon sequencing and qPCR analyses provide higher resolution into *Phoenicisalinivirus* population dynamics

**Figure 4:**
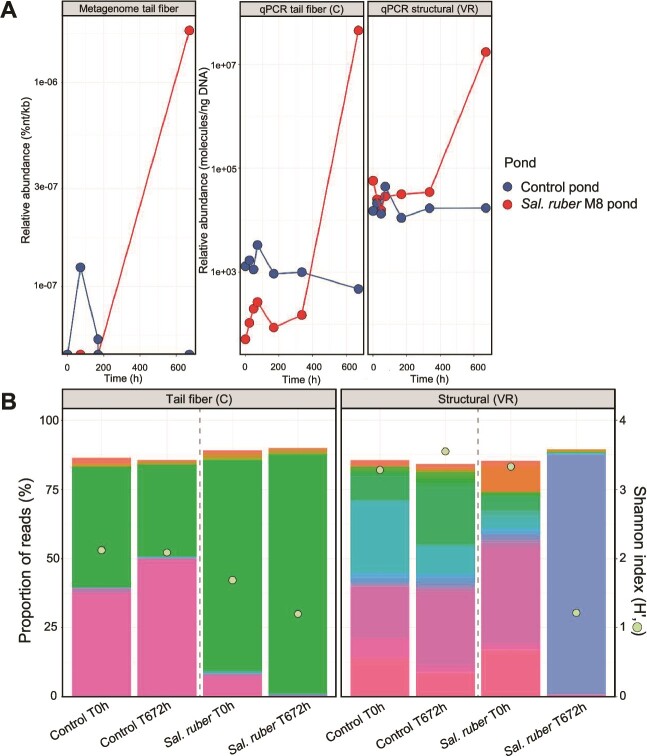
***Phoenicisalinivirus gymnesicum abundance and diversity along the experiment.*** (A) Abundance of *P. gymnesicum* in the M8 (red lines) and control (blue lines) ponds calculated as normalized percentage of the viral metagenome reads (left panel), copies per nanogram of DNA measured by qPCR with primers specific for the gene coding for the tail fiber protein (central panel), and with primers targeting a more variable region encoding for a structural protein (right panel). (B) Diversity of the tail fiber (left) and the structural protein (right) genes at times 0 h and 672 h as estimated by amplicon sequencing using extracellular viral DNA as the target and primers used in A. The bar plot represents the relative abundance of different phylotypes (in different colors) detected by amplicon sequencing measured as percentage of reads. Phylotypes were called by sequence clustering at 100% identity and coverage. A total of 57 and 77 phylotypes were identified for the tail fiber (C) and the structural protein (VR) genes, respectively. Green dots represent the Shannon index (H′).

The increase of the M8-infecting viral population was confirmed by qPCR with a primer set targeting one of the genes coding for the putative tail fiber (CCDS09 in the annotation dataset S3). Both metagenomic and qPCR analyses yielded similar patterns (Fig. 4). These results also indicate that metagenomics did not have enough sensitivity to detect this virus population initially, a phenomenon that must be kept in mind when analyzing shotgun metagenomic datasets. The higher sensitivity of qPCR compared to metagenomics has been previously documented for the detection in natural samples of specific viruses or antibiotic resistance genes [67–70]). In fact, the limit of detection of metagenomics has been estimated to be, at least, two orders of magnitude higher than that of PCR based methods, depending on the relative abundance of the targeted organism and the sequencing effort applied [71]. Thus, unless sequencing is performed at a sufficient depth, metagenomics may not be useful to distinguish between genetic evolution due to immigration, or fluctuation or selection of previously existing (but low relative abundance) genetic variants. Therefore, caution must be exerted when over-interpreting metagenomic data.

To ascertain whether there was selection of a previously available genotype or, less likely, evolution of the previously present virus, which would allow it to infect a new host, the diversity of viral genomes was tracked throughout the experiment using a amplicon sequencing approach. For this purpose, DNA from the ponds was amplified with the above mentioned primer set conveniently modified for amplicon sequencing analyses of their corresponding amplicons. A clear decrease of the diversity of the tail-fiber coding gene was observed in the amended but not in the control pond (Fig. 4B, left panel). This decrease in diversity was due to the selection of a sequence previously present in the system, which relative abundance increased only in the M8 pond and corresponded to that of the isolated viruses. Thus, the presence of strain M8 selected for a subset of viruses initially present at low concentration which carried the gene coding for the tail fiber protein that likely mediates host specificity.

For comparison, qPCR and amplicon sequencing analyses were also carried out with primers VR targeting a gene less conserved in the *P. gymnesicum* genomes (CCDS24 in Dataset S3). Most likely, as indicated by the recruitment plot and qPCR (compare qPCRs in Fig. 4A), this region was shared with other viruses initially present in the system. Amplicon sequencing analyses indicated that, at the end of the experiment, there also was a relative increase in the amended pond of a previously present sequence variant (right panel in Fig. 4B). This observation is compatible with a selection (and increase) of genomes carrying the tail fiber protein gene which would in turn select for the rest of the genes in the genome. This would explain why, at the end of the experiment, qPCRs with both primer sets yield the same concentration, while at the beginning the tail fiber coding region was less abundant.

**Figure 5:**
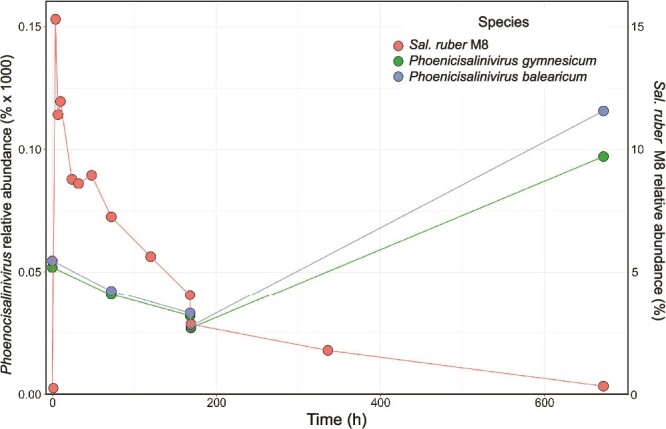
**Summary of the dynamics of *Sal. ruber* M8 and the two species of *Phoenicisalinivirus* in the amended pond along the experiment**. Relative abundance was calculated as the number of recruited nucleotides divided by metagenome size and genome size (for *Phoenicisalinivirus* values were multiplied by × 1000 to facilitate interpretation). *Sal. ruber* M8 and *Phoenicisalinivirus* relative abundance were calculated in the cellular and viral metagenomes, respectively.

Overall, metagenome, qPCR and amplicon sequencing data indicated that (i) the isolated viral populations were below the metagenomics detection limit in the system at the beginning of the experiment; (ii) their abundances increased only in the M8 amended pond, and (iii), this increase was accompanied by the selection of virus genotypes, within the original assemblage, throughout the experiment. The increase of viruses infecting *Sal. ruber* M8 in the amended pond is in good agreement with the Bank model according to which most viral genotypes are relatively rare until their hosts increase their abundance [11], and previously observed, for instance, for marine cyanophages [12]. Furthermore, the overall dynamics of *Sal. ruber* M8 and the two species of phoenicisaliniviruses does not contradict the KtW model in spite of the long delay between virus increase and host decrease. A summary of the overall changes in host and viruses is provided in Figs. 5 and S11.

The viral species-specific genomic regions of initially low coverage that were “enriched” in the M8 spiked pond corresponded to the so-called metaviromic (or metagenomic) islands. These low read recruitment regions reflect the presence of highly variable genes within co-existing variants of the same species [72] and represent areas of diversity within natural assemblages [73]. These “viral metagenomic islands” were first described in hypersaline systems [74] where they were predicted to be involved in adaptation of clonal cellular lineages, as seems to be the case for the M8 host of phoenicisaliniviruses.

The dynamic nature of these metagenomic islands is illustrated by their changes along the experiment, as shown by our time series analyses (see the comparison of the recruitment plots at different times in Fig. 3A). The species-specific region (shaded in gray and marked with an asterisk) could only be detected in the metagenomes at the end of the experiment and only in M8 pond, while it displayed a very low recruitment at the beginning. Conversely, the corresponding metagenomic island (defined as a low recruitment region) was only apparent at the beginning. Thus, sampling temporally is essential for unambiguous metagenomic island detection and full characterization of viral genomic diversity. Furthermore, when the genomes of the isolated viruses were compared, some hypervariable areas were also observed (Fig. S12). However, these areas of diversity within closely related genomes did not always correspond with metagenomics hypervariable regions (islands).

Analyses of the evolution along the experiment of the genomic regions shared by the isolates are shown in Supplementary results (“Mutation analyses”).

### *Sal. ruber* M8-*Phoenicisalinivirus* dynamics as shown by culture

*Sal. ruber* dynamics throughout the experiment was also followed by colony isolation. During the experiment, a total of 196 *Sal. ruber* strains were isolated at different time points from the pond amended with M8, 142 of which corresponded to M8 based on PCR with strain specific primers and MALDI-TOF MS identification. The strain M8 always constituted a considerable proportion of the *Sal. ruber* isolated colonies for each time point, from over 90% of the total at 2 h after inoculation to 60% at the end of the experiment. The M8-specific genomic regions (HV1 and HV2 in Fig. 1B) could not be detected in the pond by metagenomics at this later time point (when M8 specific genome recruited less than 0.4% of the metagenomics reads). Thus, the proportion of M8 among the cultured *Sal. ruber* strains was much higher than expected based on metagenomic data (compare Figs. 1G and 1H). This high cultivability could be due to M8’s adaptation to lab conditions since its isolation in 2000. In fact, in a previous work [75], metabolomic differences were found between old and new strains isolated from the same ponds that could be related to extended exposure to laboratory conditions. Alternatively, M8 may have been readily isolated at that time due to its ability to grow under lab conditions. In any case, these results highlight the complementarity of culture-based and metagenomic approaches for monitoring environmental microbial dynamics.

All 142 M8 strains isolated along the experiment were sensitive to the 10 *Phoenicivisalinivirus* representatives, whereas none of the resistant *Sal. ruber* strains corresponded to M8. Thus, M8 did not apparently develop resistance to the virus during the 28-day experiment. This is contrary to what was observed in pure liquid cultures, where resistance could be reproducibly detected after 200 h of incubation (Supplementary Fig. S13). This is reminiscent of the observation that patterns of mutation in viral genomes differ between *in vitro* and *in vivo* populations [76], and provides another example of how virus-host interactions are context dependent [2]. Furthermore, as pointed out by [77], this also highlights how the influence of the ecological context on virus-host dynamics may have profound effects on the outcomes of phage therapy.

Thus, except for *Sal. ruber* M8, no other phage-sensitive *Sal. ruber* strains could be isolated from the pond. As pointed out in Koskella et al. [1], one would have expected that *Sal. ruber* resistant strains would outcompete sensitive strains, as was indeed observed using metagenomics (see Supplementary Results for a more detailed explanation). However, this was not the case within the culturable fraction. This is a new dimension of the great plate count anomaly [78] that must be taken into account when studying virus-host interactions within an ecological context.

### *Phoenicisalinivirus* host range analyses confirmed their high specificity

Examining a series of *Sal. ruber* strains that were isolated in 2000 (8 strains) from Es Trenc and Santa Pola salterns [19], and in 2012 (37 strains) or 2019 (21 strains) from the Es Trenc salterns, showed that only two of the *Sal. ruber* strains isolated in 2012 could be infected by a phoenicisalinivirus (Supplementary Dataset S4). Furthermore, these two strains were only infected by *P. balearicum*, supporting the hypothesis that the genome region differing between the two viral species is involved in virus-host recognition. Although our numbers of isolates evaluated were too low to draw broad conclusions, these data support the previous hypothesis that hosts are more sensitive to viruses “from the future” [1], as in our case host isolated in 2012 were sensitive to viruses isolated in 2014. This can be explained by the co-evolution between viruses and host, since past hosts would have their defenses outdated [79]. Finally, the 10 *Phoenicisalinivirus* representatives were challenged against a set of 19 strains corresponding to new species of *Salinibacter* [18], which, again, were all resistant to the infection (Dataset S4). Overall, these results indicate that all *Phoenicisalinivirus* have a similar and narrow host range (they only infected strain M8), which highlights their high host specificity (except for the two *Sal. ruber* strains mentioned above which were sensitive to *P. balearicum*).

The extremely narrow host range of *Phoenicisalinivirus* is somehow unexpected since host ranges in nature are thought to be wider than predicted from lab studies [1]. One could argue that narrow host ranges are considered to be biased from the choice of host since very often, host and phages do not come from the same environment [1]. However, this is not the case here, since both viral and bacterial strains were derived from the very same samples and likely had the chance to interact and coevolve.

### Low persistence of *Phoenicisalinivirus* in the system

The phoenicisaliniviruses described herein were apparently present in the system before the addition of the external host (as shown by qPCR and amplicon sequencing analyses, Fig. 4) although their abundances in the free virus fraction were very low. This is the verification, within an ecological context, that phage density is bottom-up limited by the density of their host bacteria [2]. This is also in agreement with the fact that we could not detect a culturable co-occurring *Sal. ruber* strain throughout the experiment, other than M8, which was sensitive to these viruses. The small population sizes of phoenicisaliniviruses and their hosts in the system could have promoted the extinction of these phages unless they had undergone integration into their host genome (or a reproduction strategy other than the lytic cycle). Given that phoenicisaliniviruses harbor integrases in their genomes, this seemed a plausible hypothesis to test. In addition, the presence of prophages in the resident *Sal. ruber* strains would not only provide them with a mechanisms of resistance by superinfection exclusion, but also a mechanism of intraspecific competence with M8 [80], since prophage induction would inhibit M8 growth.

We then checked whether the 54 resistant *Sal. ruber* strains different from M8 that were isolated during the experiment (i) inhibited M8 growth on a plaque assay and (ii) harbored phoenicisaliniviruses as prophages integrated into their genomes (using PCR with specific primers). However, all results were negative. In addition, phoenicisaliniviruses could not be found as prophages in the genomes of any of the genomes from the 392 available isolated *Sal. ruber* (data not shown). Therefore, although the high salinity of the brines could contribute to the preservation of virions, how these viruses persist in the system remains a mystery.

The recruitment plot of *Phoenicisalinivirus* genomes in the viral metagenomes from Es Trenc salterns in 2019 (Fig. S14), five years after the experiment described here, indicated that the species represented by the newly isolated viruses were below the metagenomics detection limit. However, there was an assemblage of related viruses that shared some genomic regions with the phoenicisaliniviruses, as had been detected at the beginning of the experiment. This may contrast with what has been previously found in marine environments, where the same phage genomes have been found to reoccur seasonally even between biomes [81]. However, this discrepancy may be due to the different thresholds used to consider than a given genome is present in a sample. This is especially relevant considering that closely related virus genomes may have very different host ranges and infectivity and, therefore, very different ecological outcomes at the microdiversity level.

## Conclusion

As with experiments carried out with gut microbiota [76], our data show that changes in the microbiota shift the genetic diversity of bacteriophages. Along the same lines, Enav and coauthors [82] observed that the proliferation of a rare host results in an increase in the abundance of rare viral types. In our case, however, there seemed to be a selection of previously present viral genotypes and not the “generation” of new viral diversity. As noted by Berg and Roux [83], host strain diversity, not only host numbers, is a key factor controlling virus-host dynamics in natural systems. The novelty of our work is that these interactions have been unveiled in a complex environment, by leveraging an approach combining observation and manipulation of a natural system. The dynamics of the response observed here could not have been anticipated: one could have expected that the added strain disappeared without any effect in the viral assemblage (strain M8 was not present at the beginning at the limit of detection of our metagenomic effort and likely had not been present for years) or, alternatively, it could have remained as the dominant strain in the system escaping viral predation, or viruses would have had a stronger effect on the decrease of the added host. In fact, viruses increased considerably much later than the host addition, when host’s concentration was quite low. Our mixed approach has allowed “real-time” monitoring of temporal dynamics and evolution of the viral assemblages. Our results highlight how very low abundance viral genotypes can be kept “waiting” for the right host to increase their numbers, similar to seeds in a seed bank waiting for the right germination conditions. Here, only the combination of holistic (metagenomics) and reductionist (virus isolation) approaches allowed matching viral and host strains while tracking their dynamics in a natural and complex ecosystem.

## Supplementary Material

Supplementary_Figures_ALL_CORRECTED_wrae208

Ramos-Barbero_et_al_Supplementary_R_and_M_and_S_Tables_wrae208

Dataset_S1_Viral_contigs_larger_than_10_Kb_wrae208

Dataset_S2_Isolated_viruses_wrae208

Dataset_S3_Annotation_of_the_Phoenicisalinivirus_genomes_wrae208

Dataset_S4_SNP_and_mutations_wrae208

Dataset_S5_Host_range_of_the_Phoenicisaliniviruses_wrae208

Dataset_S6_Recruitments_of_Sal_ruber_strains_wrae208

FigureS15_wrae208

## References

[1] Koskella B, Hernandez CA, Wheatley RM. Understanding the impacts of bacteriophage viruses: from laboratory evolution to natural ecosystems. *Annu Rev Virol* 2022;9:57–78. 10.1146/annurev-virology-091919-075914 35584889

[2] Blazanin M, Turner PE. Community context matters for bacteria-phage ecology and evolution. *ISME J* 2021;15:3119–28. 10.1038/s41396-021-01012-x 34127803 PMC8528888

[3] Correa AMS, Howard-Varona C, Coy SR et al. Revisiting the rules of life for viruses of microorganisms. *Nat Rev Microbiol* 2021;19:501–13. 10.1038/s41579-021-00530-x 33762712

[4] Hobbs Z, Abedon ST. Diversity of phage infection types and associated terminology: the problem with ‘lytic or lysogenic’. *FEMS Microbiol Lett* 2016;363:fnw047. 10.1093/femsle/fnw047 26925588

[5] Rodriguez-Valera F, Mizuno CM, Ghai R. Tales from a thousand and one phages. *Bacteriophage* 2014;4:e28265. 10.4161/bact.28265 24616837 PMC3945994

[6] Berg M, Goudeau D, Olmsted C et al. Host population diversity as a driver of viral infection cycle in wild populations of green sulfur bacteria with long standing virus-host interactions. *ISME J* 2021;15:1569–84. 10.1038/s41396-020-00870-1 33452481 PMC8163819

[7] Di Meglio L, Santos F, Gomariz M et al. Seasonal dynamics of extremely halophilic microbial communities in three Argentinian salterns. *FEMS Microbiol Ecol* 2016;92:fiw184. 10.1093/femsec/fiw184 27604253

[8] Silveira CB, Rohwer FL. Piggyback-the-winner in host-associated microbial communities. *npj Biofilms Microbiomes* 2016;2:16010. 10.1038/npjbiofilms.2016.10 28721247 PMC5515262

[9] Chevallereau A, Pons BJ, van Houte S et al. Interactions between bacterial and phage communities in natural environments. *Nat Rev Microbiol* 2021;20:49–62. 10.1038/s41579-021-00602-y 34373631

[10] Guajardo-Leiva S, Santos F, Salgado O et al. Unveiling ecological and genetic novelty within lytic and lysogenic viral communities of hot spring phototrophic microbial mats. *Microbiol Spectr* 2021;9:e00694–21. 10.1128/Spectrum.00694-21 34787442 PMC8597652

[11] Breitbart M, Rohwer F. Here a virus, there a virus, everywhere the same virus? *Trends Microbiol* 2005;13:278–84. 10.1016/j.tim.2005.04.003 15936660

[12] Dart E, Fuhrman JA, Ahlgren NA. Diverse marine T4-like cyanophage communities are primarily comprised of low-abundance species including species with distinct seasonal, persistent, occasional, or sporadic dynamics. *Viruses* 2023;15:581. 10.3390/v15020581 36851794 PMC9960396

[13] Martinez-Hernandez F, Garcia-Heredia I, Gomez ML et al. Droplet digital PCR for estimating absolute abundances of widespread pelagibacter viruses. *Front Microbiol* 2019;10:1226. 10.3389/fmicb.2019.01226 31244789 PMC6581686

[14] Martínez Martínez J, Martinez-Hernandez F, Martinez-Garcia M. Single-virus genomics and beyond. *Nat Rev Microbiol* 2020;18:705–16. 10.1038/s41579-020-00444-0 33024311

[15] Brennan GL, Logares R. Tracking contemporary microbial evolution in a changing ocean. *Trends Microbiol* 2023;31:336–45. 10.1016/j.tim.2022.09.001 36244921

[16] Ignacio-Espinoza JC, Ahlgren NA, Fuhrman JA. Long-term stability and red queen-like strain dynamics in marine viruses. *Nat Microbiol* 2020;5:265–71. 10.1038/s41564-019-0628-x 31819214

[17] Konstantinidis KT, Viver T, Conrad RE et al. Solar salterns as model systems to study the units of bacterial diversity that matter for ecosystem functioning. *Curr Opin Biotechnol* 2022;73:151–7. 10.1016/j.copbio.2021.07.028 34438234

[18] Viver T, Conrad RE, Lucio M et al. Description of two cultivated and two uncultivated new *Salinibacter* species, one named following the rules of the bacteriological code: *Salinibacter grassmerensis* sp. nov.; and three named following the rules of the SeqCode: *Salinibacter pepae* sp. nov., *Salinibacter abyssi* sp. nov., and *Salinibacter pampae* sp. nov. *Syst Appl Microbiol* 2023;46:126416. 10.1016/j.syapm.2023.126416 36965279

[19] Antón J, Oren A, Benlloch S et al. *Salinibacter ruber* gen. Nov., sp. nov., a novel, extremely halophilic member of the bacteria from saltern crystallizer ponds. *Int J Syst Evol Microbiol* 2002;52:485–91. 10.1099/00207713-52-2-485 11931160

[20] Santos F, Yarza P, Parro V et al. The metavirome of a hypersaline environment. *Environ Microbiol* 2010;12:2965–76. 10.1111/j.1462-2920.2010.02273.x 20561021

[21] Villamor J, Ramos-Barbero MD, González-Torres P et al. Characterization of ecologically diverse viruses infecting co-occurring strains of cosmopolitan hyperhalophilic *Bacteroidetes*. *ISME J* 2018;12:424–37. 10.1038/ismej.2017.175 29099492 PMC5776456

[22] Peña A, Teeling H, Huerta-Cepas J et al. Fine-scale evolution: genomic, phenotypic and ecological differentiation in two coexisting Salinibacter ruber strains. *ISME J* 2010;4:882–95. 10.1038/ismej.2010.6 20164864

[23] Billups SC, Neville MC, Rudolph M et al. Identifying significant temporal variation in time course microarray data without replicates. *BMC bioinformatics* 2009;10:96. 10.1186/1471-2105-10-96 19323838 PMC2682797

[24] Viver T, Orellana LH, Díaz S et al. Predominance of deterministic microbial community dynamics in salterns exposed to different light intensities. *Environ Microbiol* 2019;21:4300–15. 10.1111/1462-2920.14790 31444990

[25] Antón J, Rosselló-Mora R, Rodríguez-Valera F et al. Extremely halophilic bacteria in crystallizer ponds from solar salterns. *Appl Environ Microbiol* 2000;66:3052–7. 10.1128/AEM.66.7.3052-3057.2000 10877805 PMC92110

[26] Viver T, Orellana L, González-Torres P et al. Genomic comparison between members of the Salinibacteraceae family, and description of a new species of *Salinibacter* (*Salinibacter altiplanensis* sp. nov.) isolated from high altitude hypersaline environments of the Argentinian Altiplano. *Syst Appl Microbiol* 2018;41:198–212. 10.1016/j.syapm.2017.12.004 29429564

[27] Ramos-Barbero MD, Martínez JM, Almansa C et al. Prokaryotic and viral community structure in the singular chaotropic salt Lake Salar de uyuni. *Environ Microbiol* 2019b;21:2029–42. 10.1111/1462-2920.14549 30724439

[28] Schmieder R, Edwards R. Quality control and preprocessing of metagenomic datasets. *Bioinformatics* 2011;27:863–4. 10.1093/bioinformatics/btr026 21278185 PMC3051327

[29] Peng Y, Leung HCM, Yiu SM et al. IDBA-UD: a de novo assembler for single-cell and metagenomic sequencing data with highly uneven depth. *Bioinformatics* 2012b;28:1420–8. 10.1093/bioinformatics/bts174 22495754

[30] Rodriguez-R LM, Konstantinidis KT. Nonpareil: a redundancy-based approach to assess the level of coverage in metagenomic datasets. *Bioinformatics* 2014;30:629–35. 10.1093/bioinformatics/btt584 24123672

[31] Cox MP, Peterson DA, Biggs PJ. SolexaQA : at-a-glance quality assessment of Illumina second-generation sequencing data. *BMC Bioinformatics* 2010;11:485. 10.1186/1471-2105-11-485 20875133 PMC2956736

[32] Zhu W, Lomsadze A, Borodovsky M. Ab initio gene identification in metagenomic sequences. *Nucleic Acids Res* 2010;38:e132. 10.1093/nar/gkq275 20403810 PMC2896542

[33] Altschul SF, Gish W, Miller W et al. Basic local alignment search tool. *J Mol Biol* 1990;215:403–10. 10.1016/S0022-2836(05)80360-2 2231712

[34] Su X, Pan W, Song B et al. Parallel-META 2.0: enhanced metagenomic data analysis with functional annotation, high performance computing and advanced visualization. *PLoS One* 2014;9:89323. 10.1371/journal.pone.0089323 PMC3940597 24595159

[35] Pruesse E, Peplies J, Glöckner FO. SINA: accurate high-throughput multiple sequence alignment of ribosomal RNA genes. *Bioinformatics* 2012;28:1823–9. 10.1093/bioinformatics/bts252 22556368 PMC3389763

[36] Pruesse E, Quast C, Knittel K et al. SILVA: a comprehensive online resource for quality checked and aligned ribosomal RNA sequence data compatible with ARB. *Nucleic Acids Res* 2007;35:7188–96. 10.1093/nar/gkm864 17947321 PMC2175337

[37] Ludwig W, Strunk O, Westram R et al. ARB: a software environment for sequence data. *Nucleic Acids Res* 2004;32:1363–71. 10.1093/nar/gkh293 14985472 PMC390282

[38] Mora-Ruiz MDR, Font-Verdera F, Díaz-Gil C et al. Moderate halophilic bacteria colonizing the phylloplane of halophytes of the subfamily Salicornioideae (Amaranthaceae). *Syst Appl Microbiol* 2015;38:406–16. 10.1016/j.syapm.2015.05.004 26164126

[39] Huntemann M, Ivanova NN, Mavromatis K et al. The standard operating procedure of the DOE-JGI metagenome annotation pipeline (MAP v.4). *Stand Genomic Sci* 2016;11:17. 10.1186/s40793-016-0138-x 26918089 PMC4766715

[40] Buchfink B, Xie C, Huson DH. Fast and sensitive protein alignment using DIAMOND. *Nat Methods* 2014;12:59–60. 10.1038/nmeth.3176 25402007

[41] Mulder, N. and Apweiler, R. (2007) InterPro and InterProScan. In: Bergman, N.H. (eds). Comparative Genomics. Methods in Molecular Biology™. Humana Press, Totowa, pp. 59–70, 10.1007/978-1-59745-515-2_5.

[42] The UniProt consortium . UniProt: a hub for protein information. *Nucleic Acids Res* 2015;43:D204–12. 10.1093/nar/gku989 25348405 PMC4384041

[43] Ondov BD, Treangen TJ, Melsted P et al. Mash: fast genome and metagenome distance estimation using MinHash. *Genome Biol* 2016;17:132. 10.1186/s13059-016-0997-x 27323842 PMC4915045

[44] Ulyantsev VI, Kazakov SV, Dubinkina VB et al. MetaFast: fast reference-free graph-based comparison of shotgun metagenomic data. *Bioinformatics* 2016;32:2760–7. 10.1093/bioinformatics/btw312 27259541

[45] Moraru C, Varsani A, Kropinski AM. VIRIDIC-A novel tool to calculate the intergenomic similarities of prokaryote-infecting viruses. *Viruses* 2020;12:1268. 10.3390/v12111268 33172115 PMC7694805

[46] Rodriguez-R LM, Konstantinidis KT. The enveomics collection: a toolbox for specialized analyses of microbial genomes and metagenomes. *PeerJ Prepr* 2016. 10.7287/peerj.preprints.1900v1

[47] Sullivan MJ, Petty NK, Beatson SA. Easyfig: a genome comparison visualizer. *Bioinformatics* 2011;27:1009–10. 10.1093/bioinformatics/btr039 21278367 PMC3065679

[48] Nishimura Y, Yoshida T, Kuronishi M et al. ViPTree: the viral proteomic tree server. *Bioinformatics* 2017;33:2379–80. 10.1093/bioinformatics/btx157 28379287

[49] Li W, Godzik A. Cd-hit: a fast program for clustering and comparing large sets of protein or nucleotide sequences. *Bioinformatics* 2006;22:1658–9. 10.1093/bioinformatics/btl158 16731699

[50] Richter M, Rosselló-Móra R, Oliver Glöckner F et al. JSpeciesWS: a web server for prokaryotic species circumscription based on pairwise genome comparison. *Bioinformatics* 2016;32:929–31. 10.1093/bioinformatics/btv681 26576653 PMC5939971

[51] Metsalu T, Vilo J. ClustVis: a web tool for visualizing clustering of multivariate data using principal component analysis and heatmap. *Nucleic Acids Res* 2015;43:W566–70. 10.1093/nar/gkv468 25969447 PMC4489295

[52] Bolger AM, Lohse M, Usadel B. Trimmomatic: a flexible trimmer for Illumina sequence data. *Bioinformatics* 2014;30:2114–20. 10.1093/bioinformatics/btu170 24695404 PMC4103590

[53] Magoč T, Salzberg SL. FLASH: fast length adjustment of short reads to improve genome assemblies. *Bioinformatics* 2011;27:2957–63. 10.1093/bioinformatics/btr507 21903629 PMC3198573

[54] McMurdie PJ, Holmes S. Phyloseq: an R package for reproducible interactive analysis and graphics of microbiome census data. *PLoS One* 2013;8:e61217. 10.1371/journal.pone.0061217 23630581 PMC3632530

[55] Wickham H . ggplot2: Elegant Graphics for Data Analysis. New York Retrieved from https://ggplot2.tidyverse.org: Springer-Verlag, 2016, 10.1007/978-3-319-24277-4

[56] Castro JC, Rodriguez-R LM, Harvey WT et al. imGLAD: accurate detection and quantification of target organisms in metagenomes. *PeerJ* 2018;6:e5882. 10.7717/peerj.5882 30405973 PMC6216955

[57] Connell JH . The influence of interespecific competition and other factors on the distribution of the barnacle *Chthamalus stellatus*. *Ecology* 1961;42:710–23. 10.2307/1933500

[58] Adriaenssens E, Brister JR. How to name and classify your phage: an informal guide. *Viruses* 2017;9:70. 10.3390/v9040070 28368359 PMC5408676

[59] Adriaenssens EM, Roux S, Brister JR et al. Guidelines for public database submission of uncultivated virus genome sequences for taxonomic classification. *Nat Biotechnol* 2023;41:898–902. 10.1038/s41587-023-01844-2 37430074 PMC10526704

[60] Turner D, Adriaenssens EM, Tolstoy I et al. Phage annotation guide: guidelines for assembly and high-quality annotation. *Phage* 2021;2:170–82. 10.1089/phage.2021.0013 35083439 PMC8785237

[61] Pepin KM, Domsic J, McKenna R. Genomic evolution in a virus under specific selection for host recognition. *Infect Genet Evol* 2008;8:825–34. 10.1016/j.meegid.2008.08.008 18804189

[62] Martinez-Hernandez F, Fornas O, Lluesma Gomez M et al. Single-virus genomics reveals hidden cosmopolitan and abundant viruses. *Nat Commun* 2017;8:15892. 10.1038/ncomms15892 28643787 PMC5490008

[63] Ramos-Barbero MD, Martin-Cuadrado A-B, Viver T et al. Recovering microbial genomes from metagenomes in hypersaline environments: the good, the bad and the ugly. *Syst Appl Microbiol* 2019a;42:30–40. 10.1016/j.syapm.2018.11.001 30528276

[64] Meziti A, Tsementzi D, Rodriguez-R LM et al. Quantifying the changes in genetic diversity within sequence-discrete bacterial populations across a spatial and temporal riverine gradient. *ISME J* 2019;13:767–79. 10.1038/s41396-018-0307-6 30397261 PMC6461791

[65] Roux S, Adriaenssens EM, Dutilh BE et al. Minimum information about an uncultivated virus genome (MIUViG). *Nat Biotechnol* 2018;37:29–37. 10.1038/nbt.4306 30556814 PMC6871006

[66] Nobrega FL, Vlot M, de Jonge PA et al. Targeting mechanisms of tailed bacteriophages. *Nat Rev Microbiol* 2018;16:760–73. 10.1038/s41579-018-0070-8 30104690

[67] Blanco-Picazo P, Gómez-Gómez C, Aguiló-Castillo S et al. Chicken liver is a potential reservoir of bacteriophages and phage-derived particles containing antibiotic resistance genes. *Microb Biotechnol* 2022;15:2464–75. 10.1111/1751-7915.14056 35485188 PMC9437878

[68] Fernández-Orth D, Miró E, Brown-Jaque M et al. Faecal phageome of healthy individuals: presence of antibiotic resistance genes and variations caused by ciprofloxacin treatment. *J Antimicrob Chemother* 2019;74:854–64. 10.1093/jac/dky540 30649322

[69] Ferreira C, Otani S, Aarestrup FM et al. Quantitative PCR versus metagenomics for monitoring antibiotic resistance genes: balancing high sensitivity and broad coverage. *FEMS Microbes* 2023;4:xtad008. 10.1093/femsmc/xtad008 37333442 PMC10117749

[70] Hjelmsø MH, Hellmér M, Fernandez-Cassi X et al. Evaluation of methods for the concentration and extraction of viruses from sewage in the context of metagenomic sequencing. *PLoS One* 2017;12:e0170199. 10.1371/journal.pone.0170199 28099518 PMC5242460

[71] Lindner BG, Suttner B, Zhu KJ et al. Toward shotgun metagenomic approaches for microbial source tracking sewage spills based on laboratory mesocosms. *Water Res* 2022;210:117993. 10.1016/j.watres.2021.117993 34979467

[72] Bellas CM, Schroeder DC, Edwards A et al. Flexible genes establish widespread bacteriophage pan-genomes in cryoconite hole ecosystems. *Nat Commun* 2020;11:4403. 10.1038/s41467-020-18236-8 32879312 PMC7468147

[73] Mizuno CM, Ghai R, Rodriguez-Valera F. Evidence for metaviromic islands in marine phages. *Front Microbiol* 2014;5:27. 10.3389/fmicb.2014.00027 24550898 PMC3909814

[74] Garcia-Heredia I, Martin-Cuadrado A-B, Mojica FJM et al. Reconstructing viral genomes from the environment using fosmid clones: the case of haloviruses. *PLoS One* 2012;7:e33802. 10.1371/journal.pone.0033802 22479446 PMC3316494

[75] Antón J, Lucio M, Peña A et al. High metabolomic microdiversity within co-occurring isolates of the extremely halophilic bacterium *Salinibacter ruber*. *PLoS One* 2013;8:e64701. 10.1371/journal.pone.0064701 23741374 PMC3669384

[76] De Sordi L, Khanna V, Debarbieux L. The gut microbiota facilitates drifts in the genetic diversity and infectivity of bacterial viruses. *Cell Host Microbe* 2017;22:801–808.e3. 10.1016/j.chom.2017.10.010 29174401

[77] Mumford R, Friman VP. Bacterial competition and quorum sensing signalling shape the eco-evolutionary outcomes of model in vitro phage therapy. *Evol Appl* 2017;10:161–9. 10.1111/eva.12435 28127392 PMC5253424

[78] Harwani D . The great plate count anomaly and the unculturable bacteria. *Int J Sci Res* 2012;2:350–1. 10.15373/22778179/SEP2013/122

[79] Gómez P, Ashby B, Buckling A. Population mixing promotes arms race host-parasite coevolution. *R Soc* 2014;282:20142297. 10.1098/rspb.2014.2297 PMC4262181 25429018

[80] Roossinck MJ . The good viruses: viral mutualistic symbioses. *Nat Rev Microbiol* 2011;9:99–108. 10.1038/nrmicro2491 21200397

[81] Aylward FO, Boeuf D, Mende DR et al. Diel cycling and long-term persistence of viruses in the ocean’s euphotic zone. *Proc Natl Acad Sci USA* 2017;114:11446–51. 10.1073/pnas.1714821114 29073070 PMC5663388

[82] Enav H, Kirzner S, Lindell D et al. Adaptation to sub-optimal hosts is a driver of viral diversification in the ocean. *Nat Commun* 2018;9:4698. 10.1038/s41467-018-07164-3 30409965 PMC6224464

[83] Berg M, Roux S. Extreme dimensions—how big (or small) can tailed phages be? *Nat Rev Microbiol* 2021;19:407. 10.1038/s41579-021-00574-z 33981030

