# Supplementary material for: Experimental evolution at ecological scales allows linking of viral genotypes to specific host strains
Source: ISME J. 2024 Nov 23;18(1):wrae208. doi: 10.1093/ismejo/wrae208 (PMC11631230; doi:10.1093/ismejo/wrae208)
Supplement: Supplementary_Figures_ALL_CORRECTED_wrae208 [file supplementary_figures_all_corrected_wrae208.pdf]

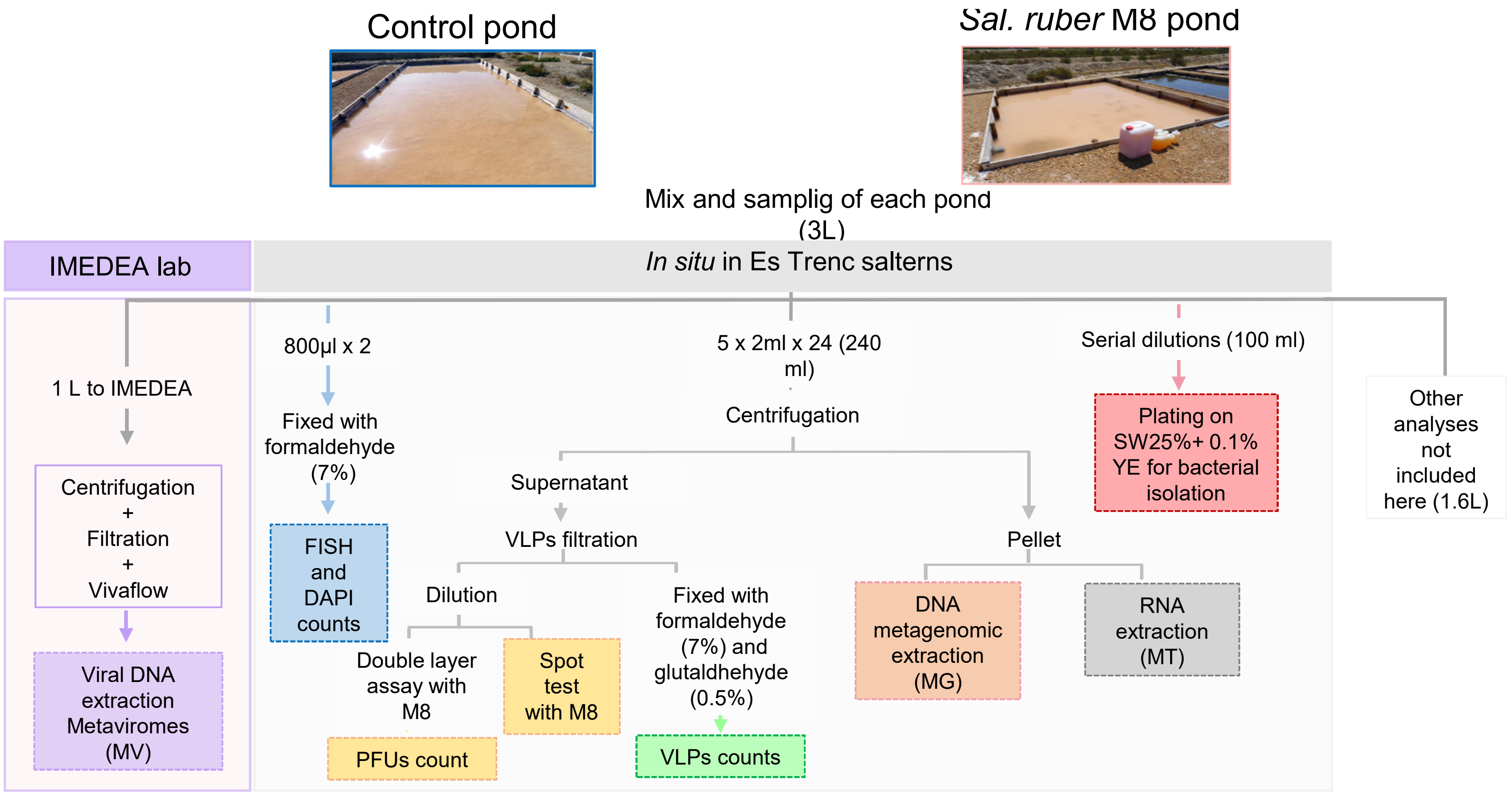

Time series of sampling (hours)

	0	4	7	10	24	32	48	72	120	168	169	360	672
DAPI/FISH													
VLPs													
MG													
MV													
MT													
Plaque													
Culture													

Figure S1. Experimental approach. After mixing the brine in the pond (as shown in figure 1) taken care not to disrupt the bottom salt crust, 3 liters of sample were taken. One liter was immediately kept On ice and brought by car to the laboratory in IMEDEA for purification and concentration of the virus assemblage; the rest of the sample manipulations (cell pelleting for DNA extraction, plating for bacteria isolation and double layers for initial virus isolation by plaque assay) were carried out in the field laboratory set for the experiment (lower insert). The table at the bottom shows the times of sampling (in hours) together with the analyses carried out with the samples. Cells, Archaea, Bacteria (DAPI/FISH) and viruses (VLPs) were measured in all samples. Colors in the table refer to the activities in the experimental approach.

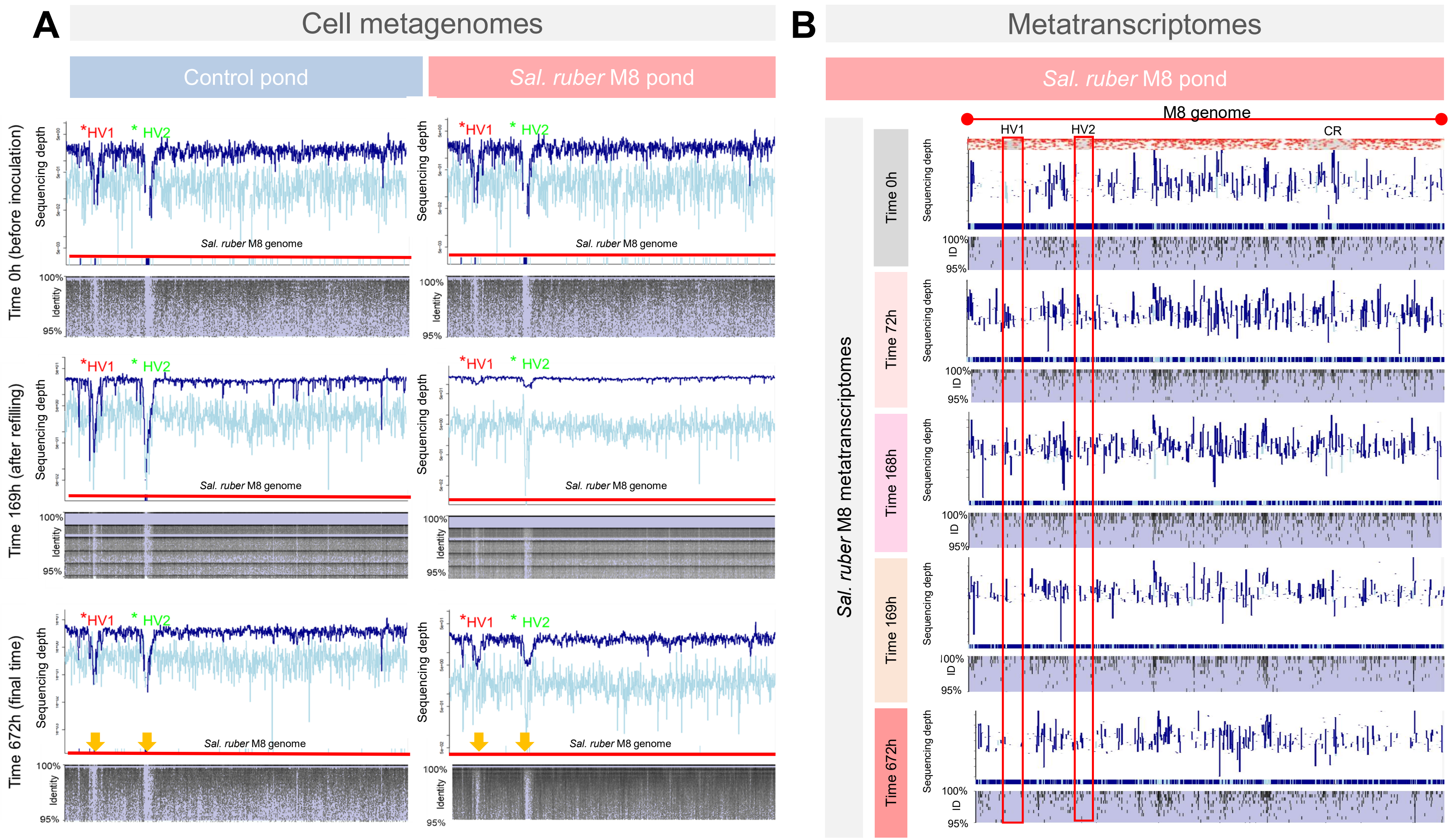

Figure S2. Evaluation of *Salinibacter ruber* M8 presence during the experiment.(A) Recruitment plots of *Sal. ruber* M8 genomes in the metagenomes of the control (left panels) and the *Sal. ruber* M8 pond (right panels). This recruitment plots illustrates the results of a BLASTn search of metagenomic reads (each matching read is represented by a black dot in purple panel) against a *Sal. ruber* M8 genome (line red in X-axis). The *Sal. ruber* M8 hypervariability islands are indicate as HV in X-axis, the red one corresponds with HV1 and the green one is the HV2. Dark and light blues lines on the top show the sequencing depth across the *Sal. ruber* M8 genome on a logarithmic scale. The dark ones indicate the sequencing depth from 95 to 100% of identity and the light one below 95% of identity. Bars at the bottom indicate regions without mapping reads (sequencing depth of zero). Orange arrows indicate the hypervariablility island recruitment in time 672h. Y-axis represents the identity range of mapped reads (B) *Sal. ruber* M8 pond metatranscriptomes recruited against *Sal. ruber* M8 complete genome. Y- axis corresponds with identity range of metratranscriptomic reads and X-axis (red dot line) shows the position of of *Sal. ruber* M8 hypervariable regions and conserved region (CR) previously described by Peña et al. 2010. The hypervariable islands HV1 and HV2 recruitment in each time is highlighted with a red box.

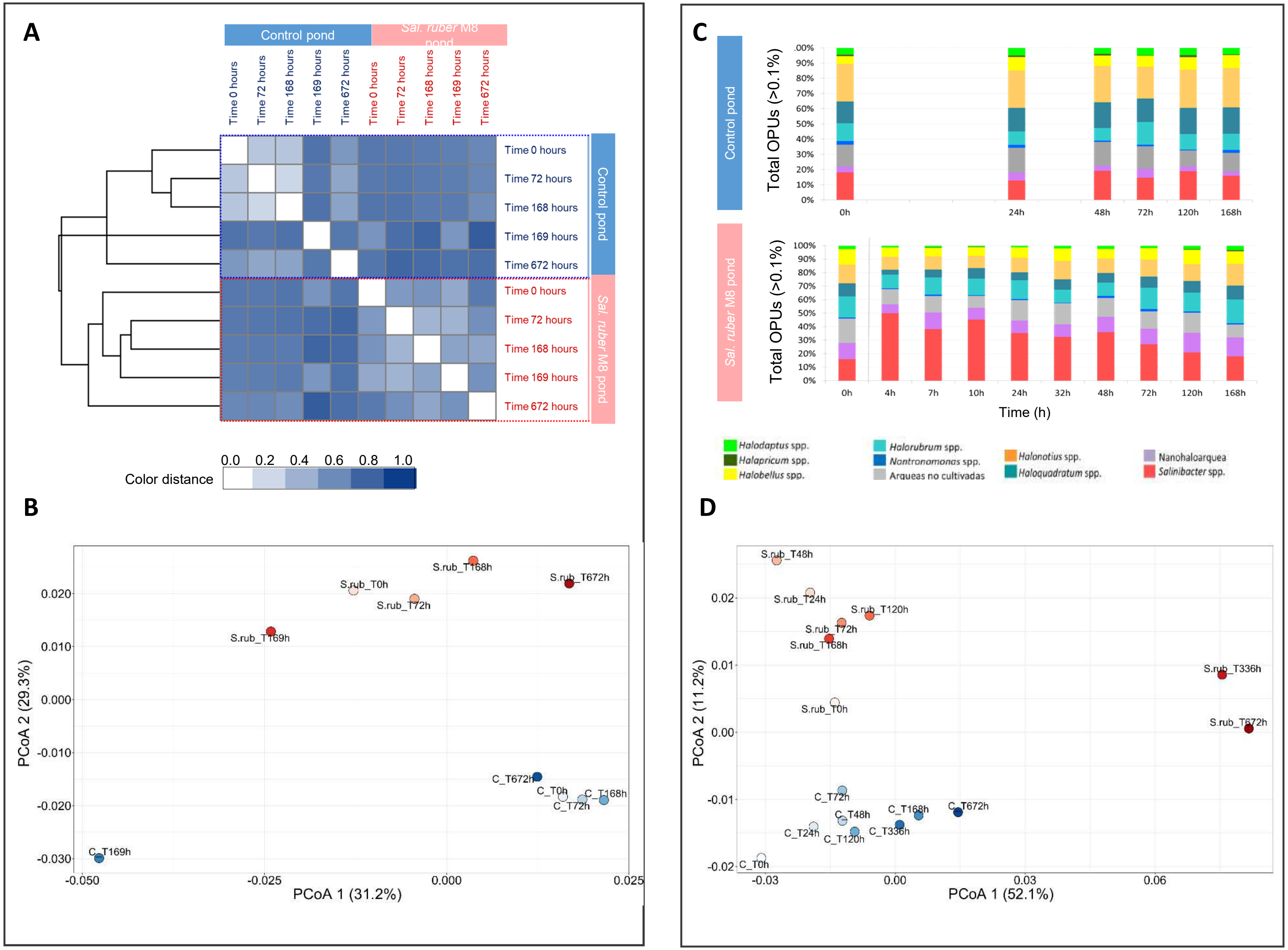

Figure S3. Dynamic of the viral (A and B) and cellular (C and D) communities along the experiment in the control and the amended pond. (A) Metafast distances in the viral community (B) PCoA of viral metagenomes based on MASH distances. (C) Taxonomic assignment of 16S rRNA based OPUs from metagenomics reads and (D) PCoA of cellular metagenomes distances. Blue dots correspond to the control pond and red-pink dots to the M8 pond. For each color, the gradient in color intensity indicates the sampling time, from the beginning (lighter) to the end (darker) of the experiment.

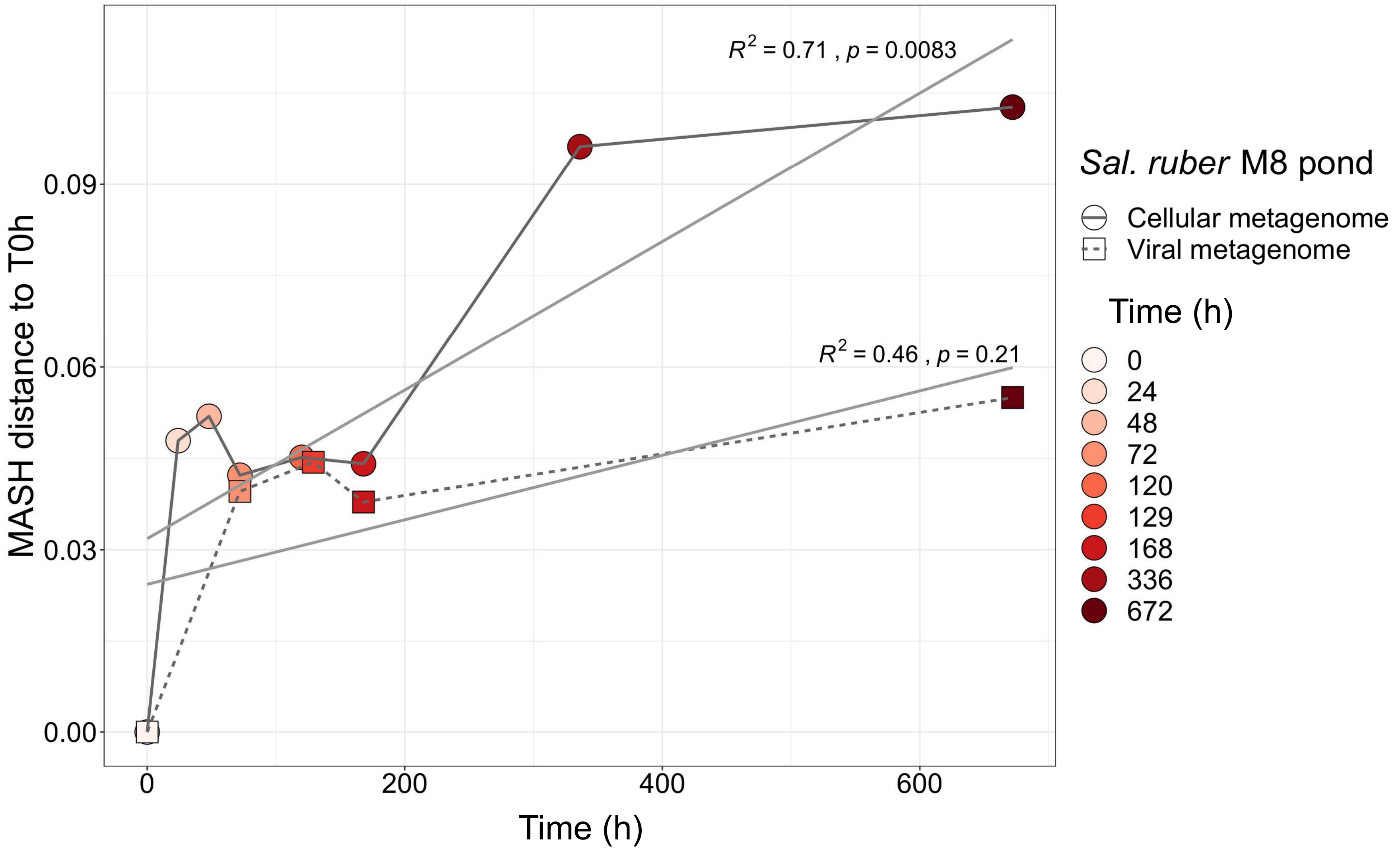

Figure S4. Comparison of MASH distances for the different time points with the time zero community in the amended pond for the cellular (circles) and viral (squares) assemblages. While the hypothesis that for the viral assemblage the distances are constant can not be rejected (p-value 0.21), it can be concluded that distances increase from time zero for the cellular assemblage (P-value 0.0083).

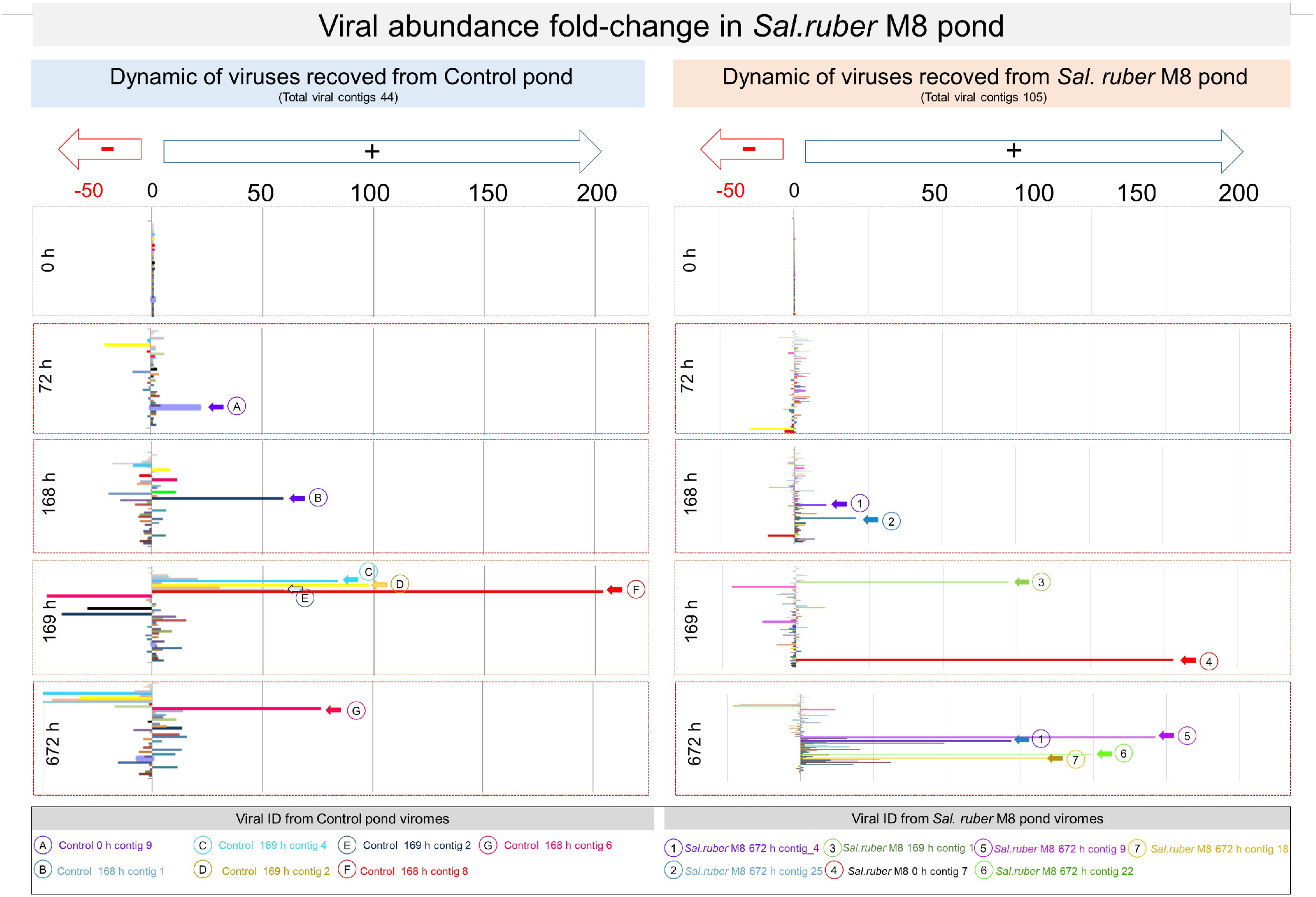

Figure S5. Fold change abundance of viral community in *Sal. ruber* M8 pond. Graphs show the fold change abundance of viral contigs larger than 10 Kb recovered from Control pond (left panel) and *Sal. ruber* M8 pond (right panel) in all times of *Sal. ruber* M8 pond. The fold change was measured as the variation in abundance at each time point compared to the initial time (0 h) in the *Sal. ruber* M8 pond. The viral contig with highest abundance fold change are labeled with a color circle in the graph. Viral genome recovered from Control pond are label with a letters and in other hand the viral genomes from *S. ruber* M8 pond are labeled with number. Among them, only 14 showed a relative abundance pattern that could be related to the decrease of *Sal. ruber* M8 strain. The contigs that increased at 169 hours were excluded from further analysis as we could not ascertain their origin. However, except their relatively high GC content, genomic analyses did not unveil any trait (e.g. CRISPR protospacers, tRNAs, etc.) that could unambiguously identify them as infecting *Sal. ruber*. This was also confirmed by the taxonomic assignment of the annotated genes, which largely corresponded to haloarchaea and their viruses. In addition, GC content did not increase in the viral metagenome from the *Sal. ruber* M8 pond (Table 1), as one would have expected if an increase of *Sal. ruber* viruses had occurred. Viral genome ID indicates viral origin (*Sal. ruber* or Control pond), virome time in hours and contig number.

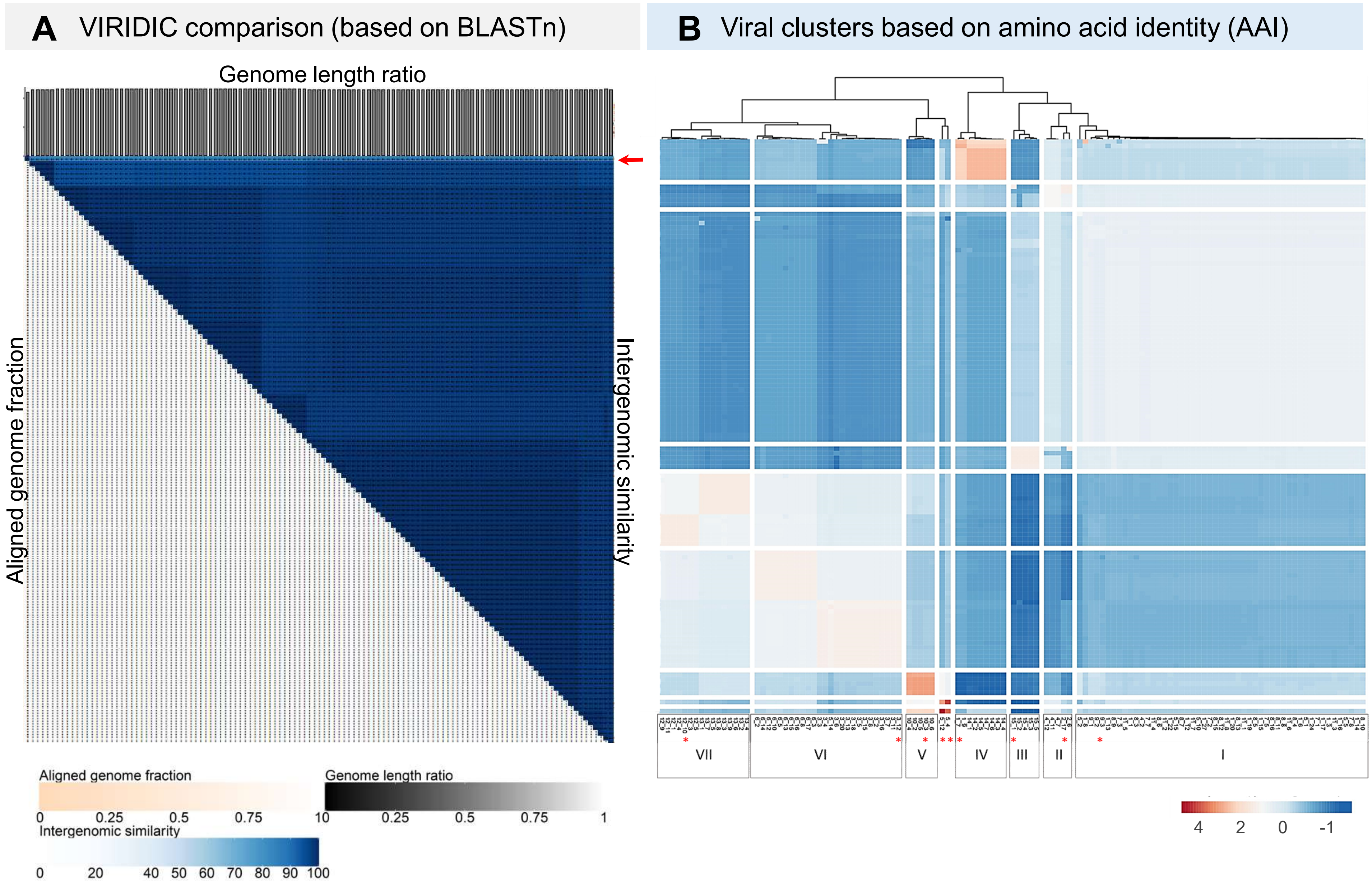

Figure S6. (A) VIRIDIC comparison (based on BLASTn) of the 120 non redundant viral genomes isolated in the experiment showing that they constitute two viral species according to intergenomic similarity (colored according to the scale at the bottom). A threshold of 95% intergenomic similarity was chosen for species delineation, according to the ICVT criteria. The arrow points to virus B2_17 (left panel). (B) Viral clusters based on amino acid identity (AAI) of the *P. gimnesicum* isolates. Groups of viral genomes cluster in the dendrogram based on the dissimilarity matrix and are colored according to the distance between groups (scale on the bottom right); representatives of groups are marked with red asterisks (right panel).

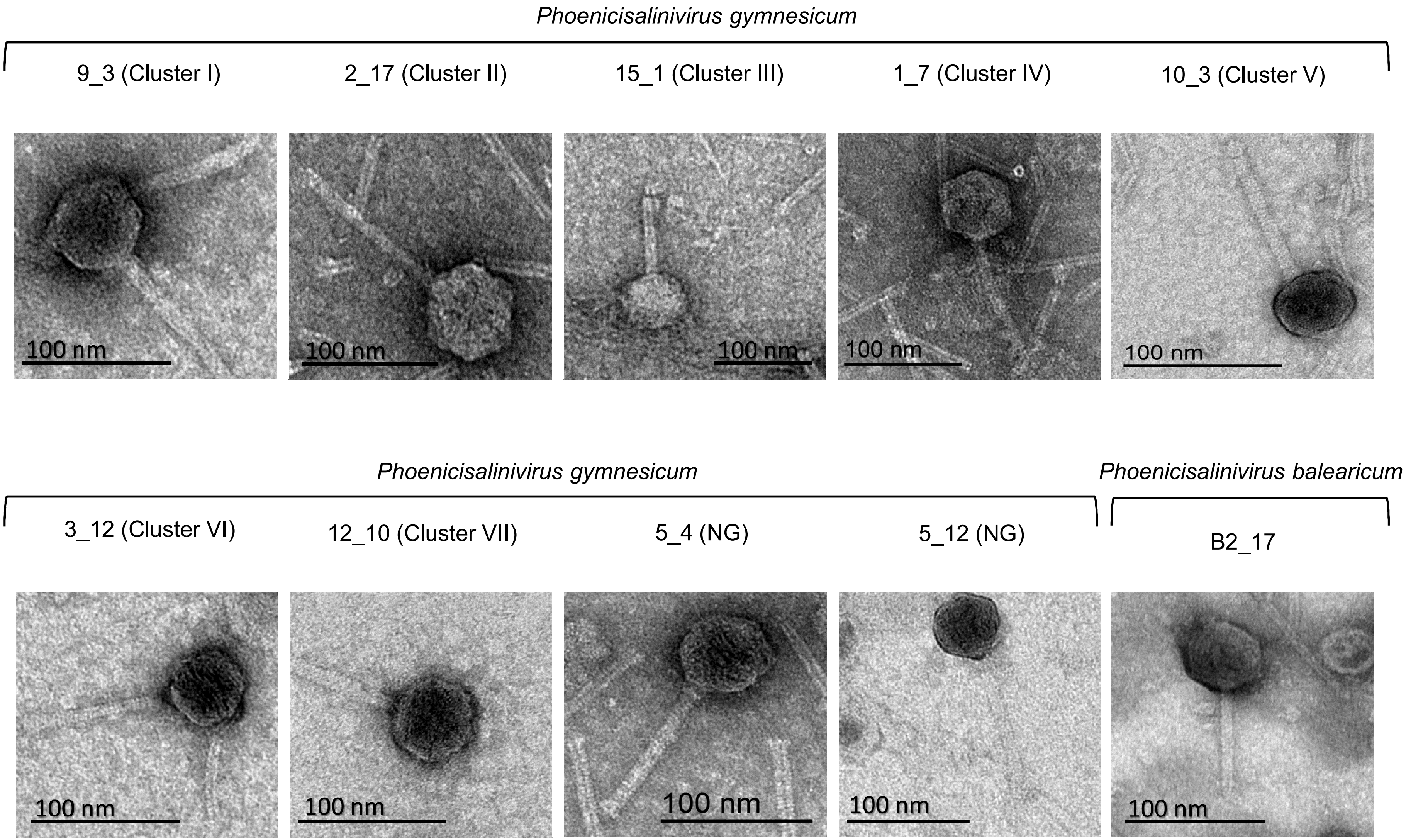

Figure S7. Negative stain of M8 isolated viruses visualized by transmission electronic microscopy. The pictures include the 9 representatives of *Phoenicisalinivirus gymnesicum* and the only representative of the *P. balearicum* viral species.

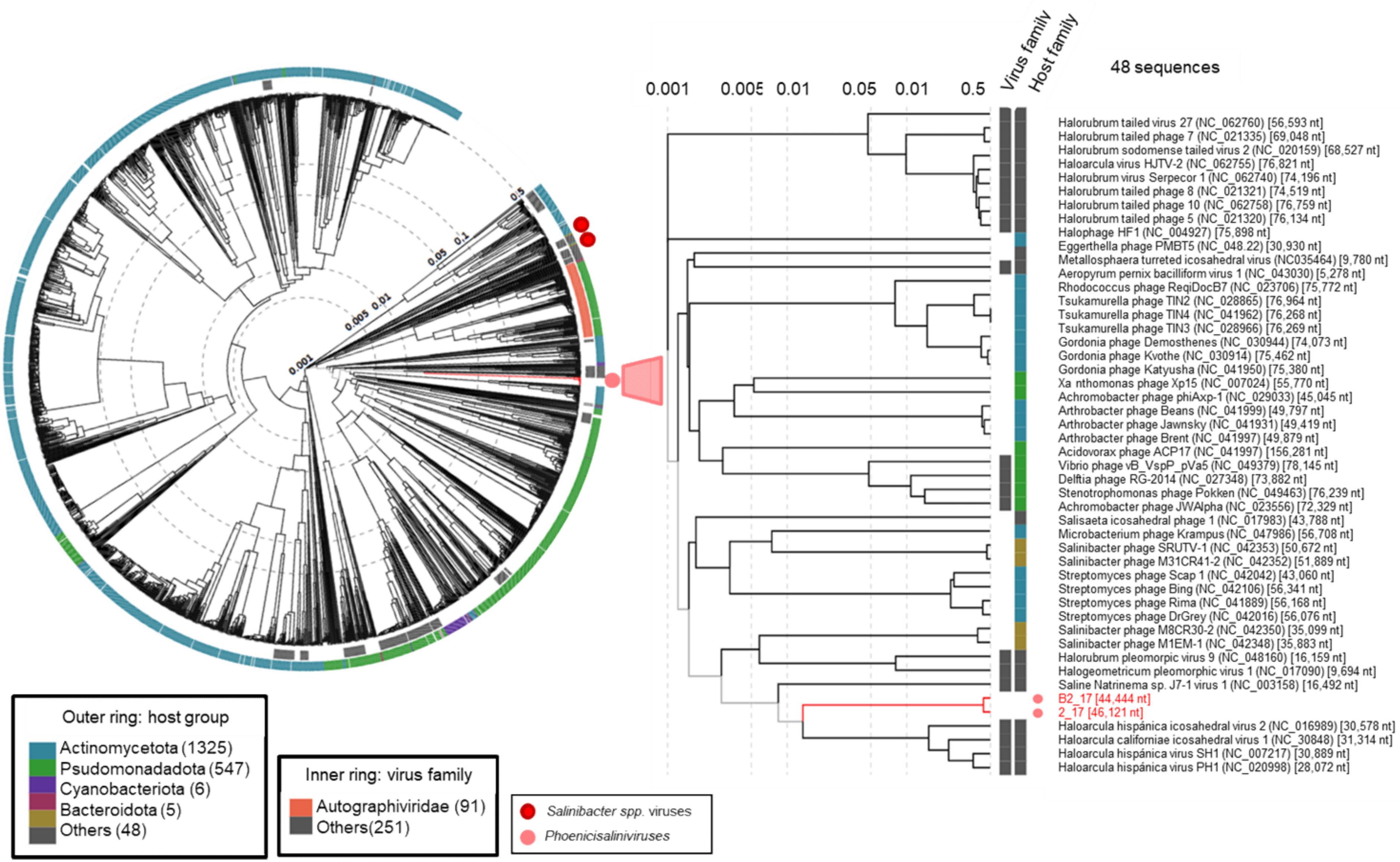

Figure S8. Proteomic tree calculated with VIPTREE of representatives of the two *Phoenicisalinivirus* species. Left panel: overview of the complete tree showing the position of the previously isolated viruses infecting *Sal. ruber* (red dots). Right panel: a closer look at the position of *Phoenicisaliniviruses* in the tree.

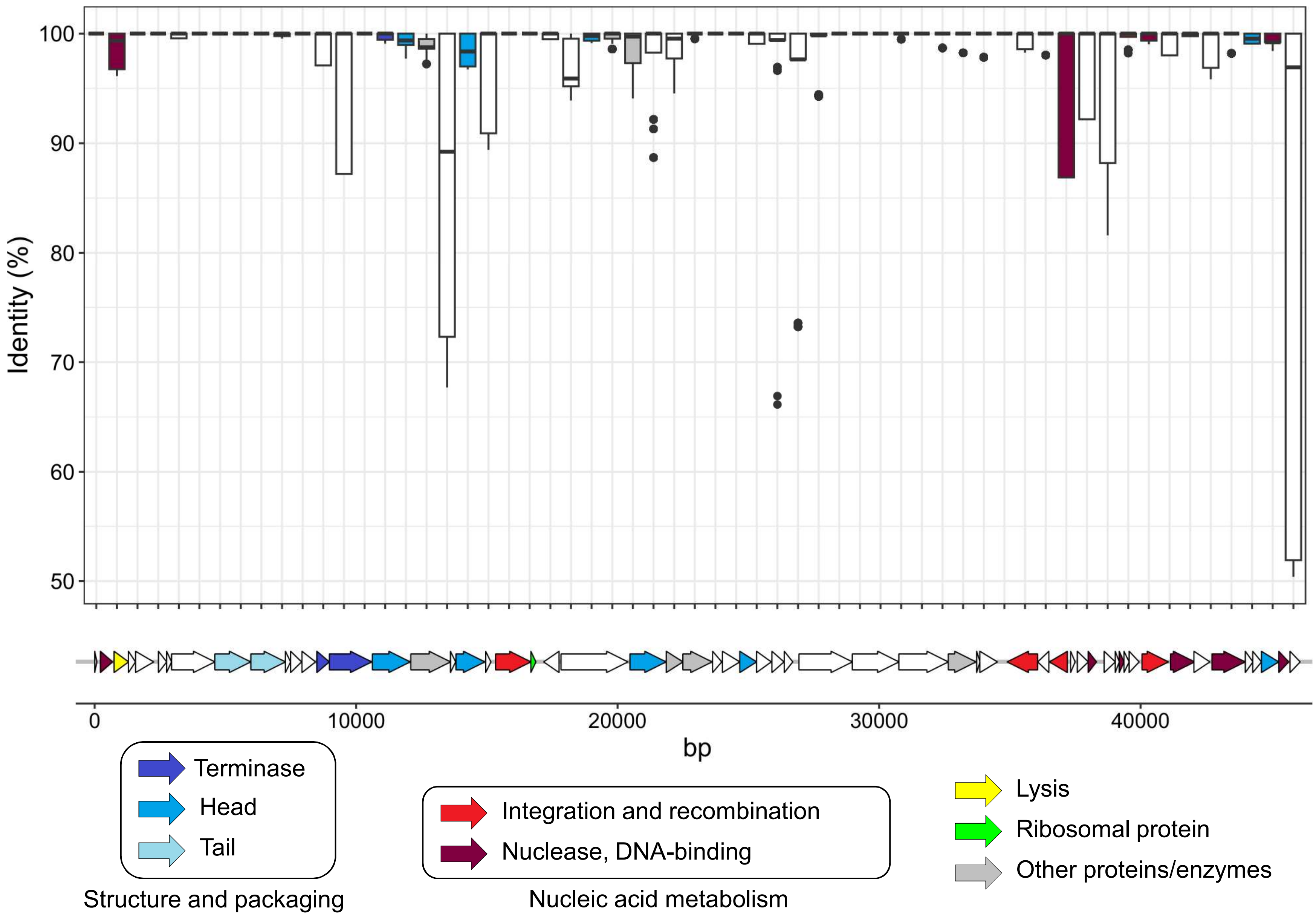

Figure S9. Diversity of *Phoenecisalinivirus* isolates. At the top, boxplot of the identity between the reciprocal best match genes of the 9 representative genomes of *P. gimnesicum*. Each boxplot represent a gene and they are ordered as they appear in virus 9_3, represented at the bottom. Color indicates the function (see legend).

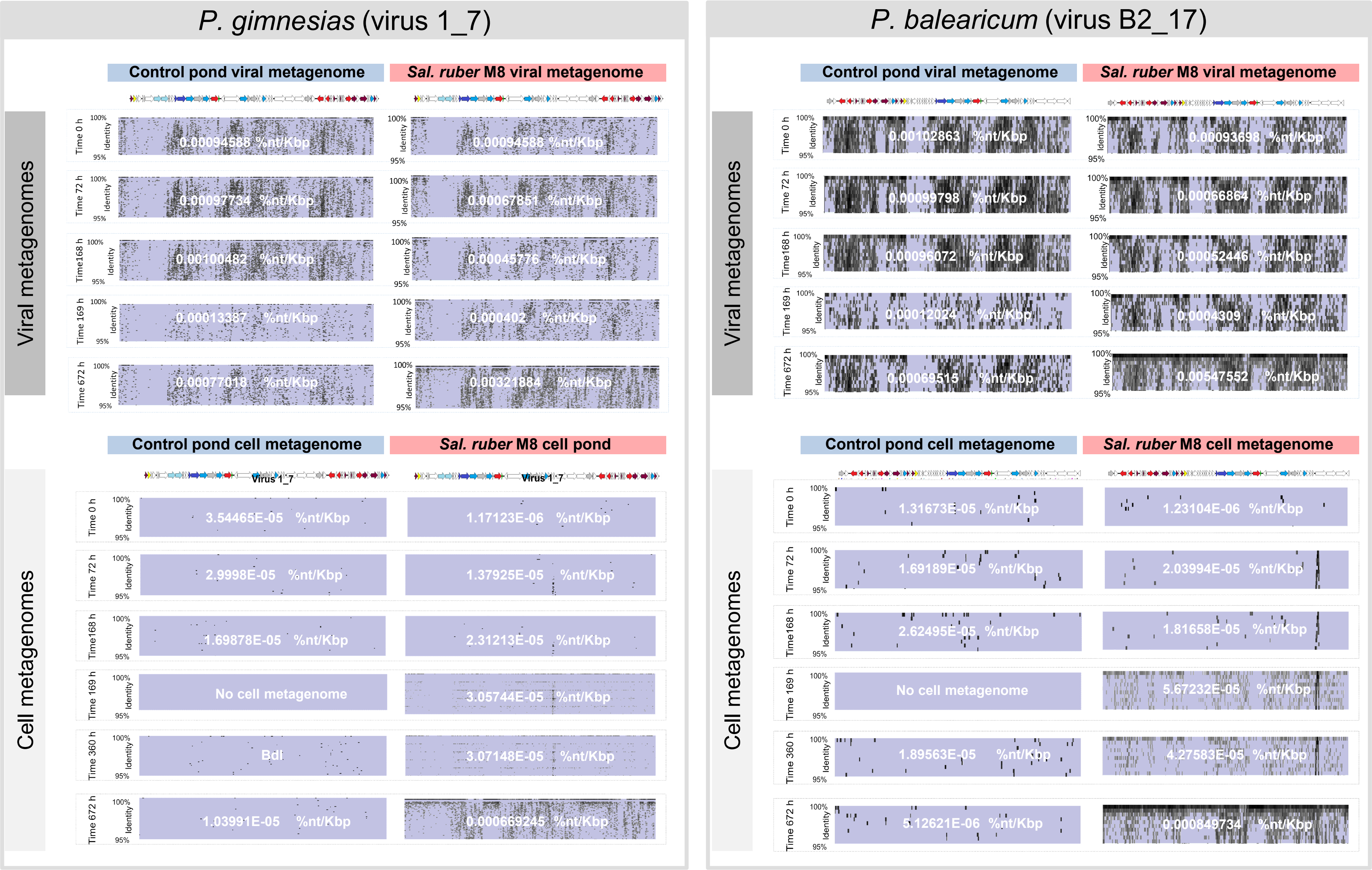

Figure S10. Dynamics of the two *Phoenicisalinivirus* species in the control pond (blue background) and the M8 amended (red background) pond. The recruitment of the genomes against the cellular (MG) and the viral (MV) metagenomes is shown. Left panel: recruitment of *P. gimnesicum.* Right panel: *P. balearicum*. The percentage of normalized recruited reads are indicated for each recruitment plot.

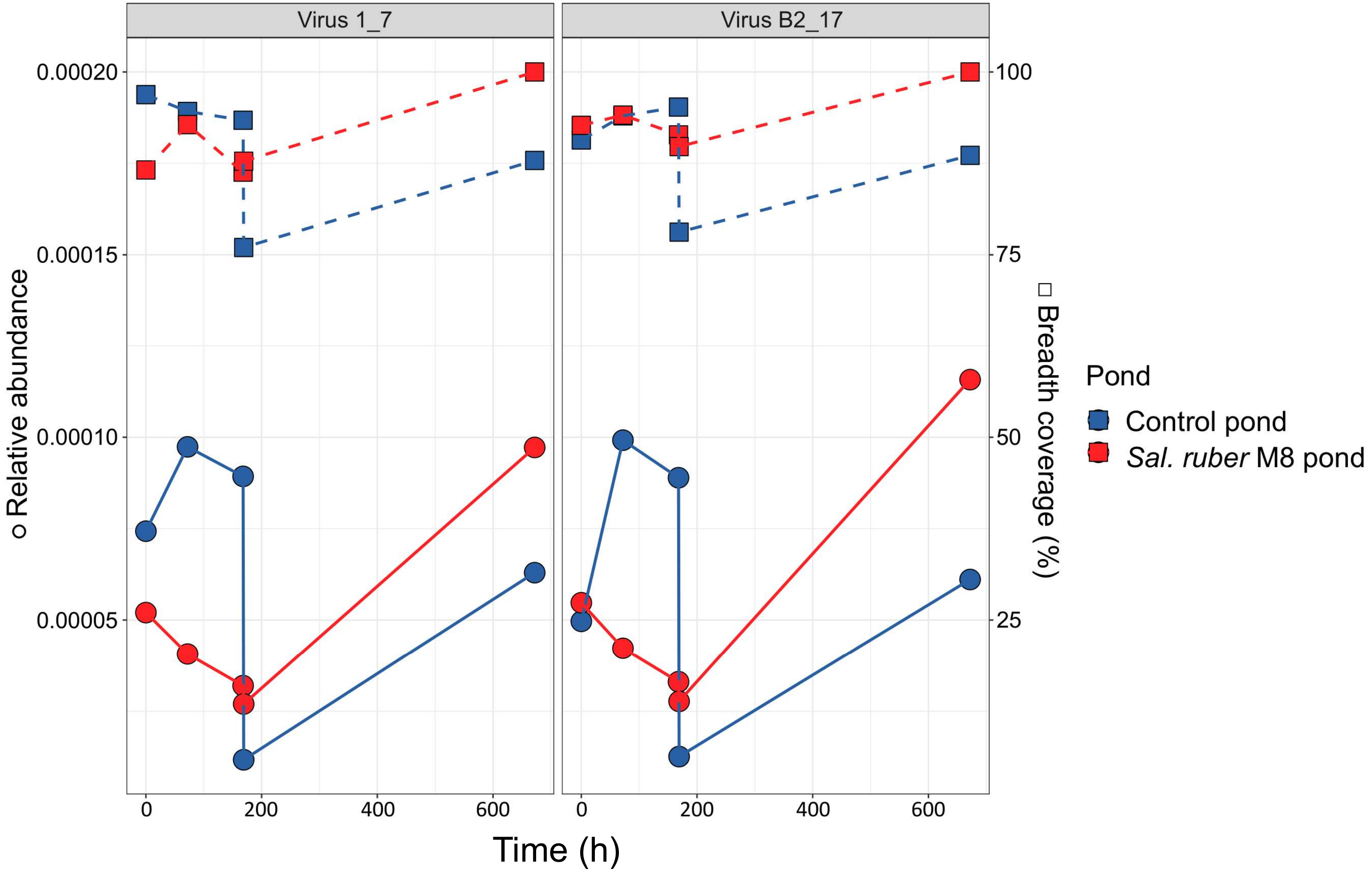

Figure S11. Viral abundance and genome coverage of *P. gymnesicum* (virus 1_7) and *P. balearicum* (virus B2_17) in control and *Sal. ruber* ponds. The left Y-axis represents the relative abundance (% nucleotides/Kbp/Gbp) of both viral species over the course of the experiment. The right Y-axis shows the horizontal genome coverage detected for each species during the experiment

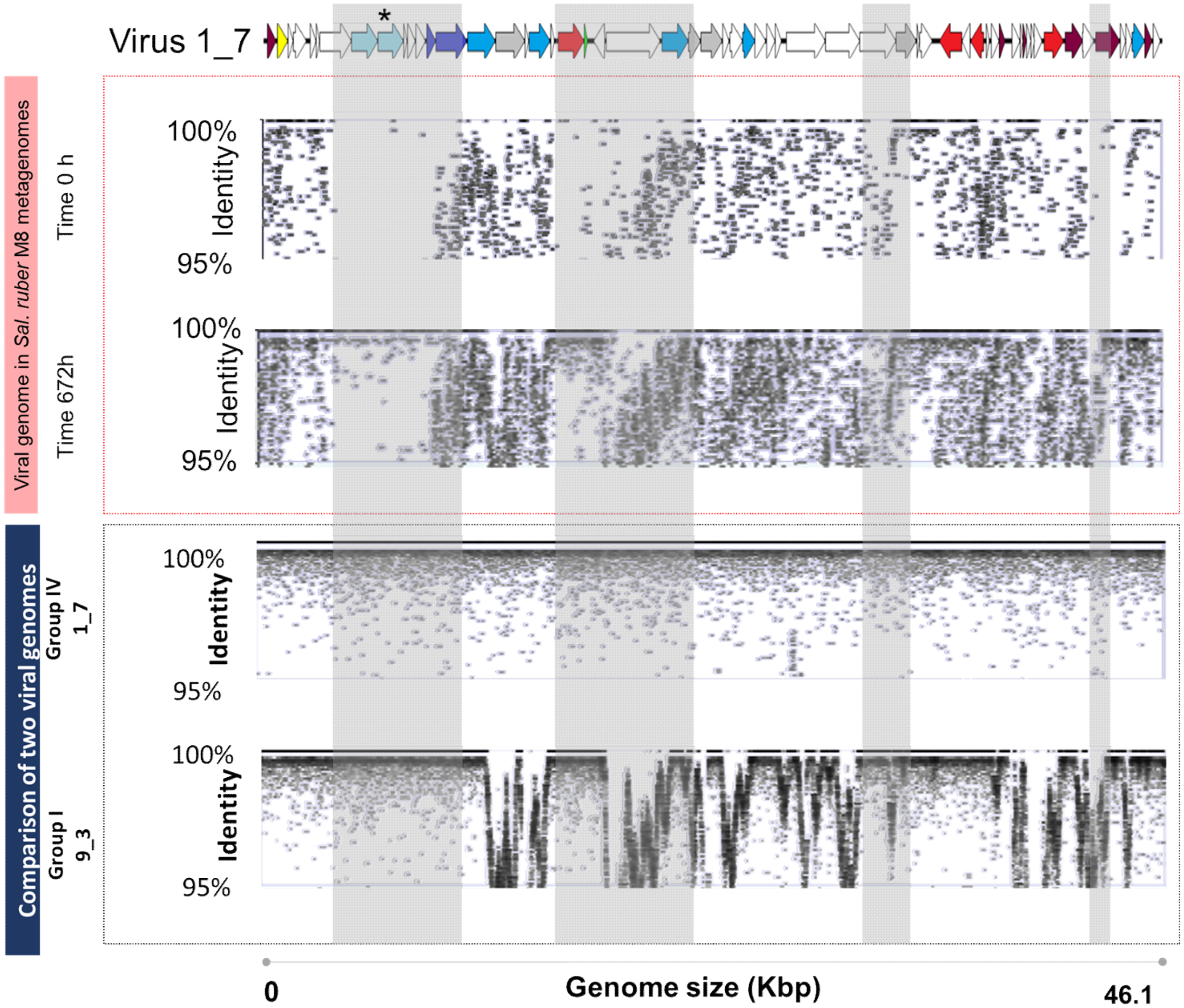

Figure S12. Comparison of metagenomic and genomic islands for *P. gimnesicum*. Upper panel: viral metagenomic read recruitment against one *P. gimnesicum* genome at the beginning and the end of the experiment (the island including tail fiber coding genes is marked with an asterisk). Lower panel: comparison of two viral genomes within the virus family (reads for each genomic sequence are recruited against virus 1_7 assembled genome).

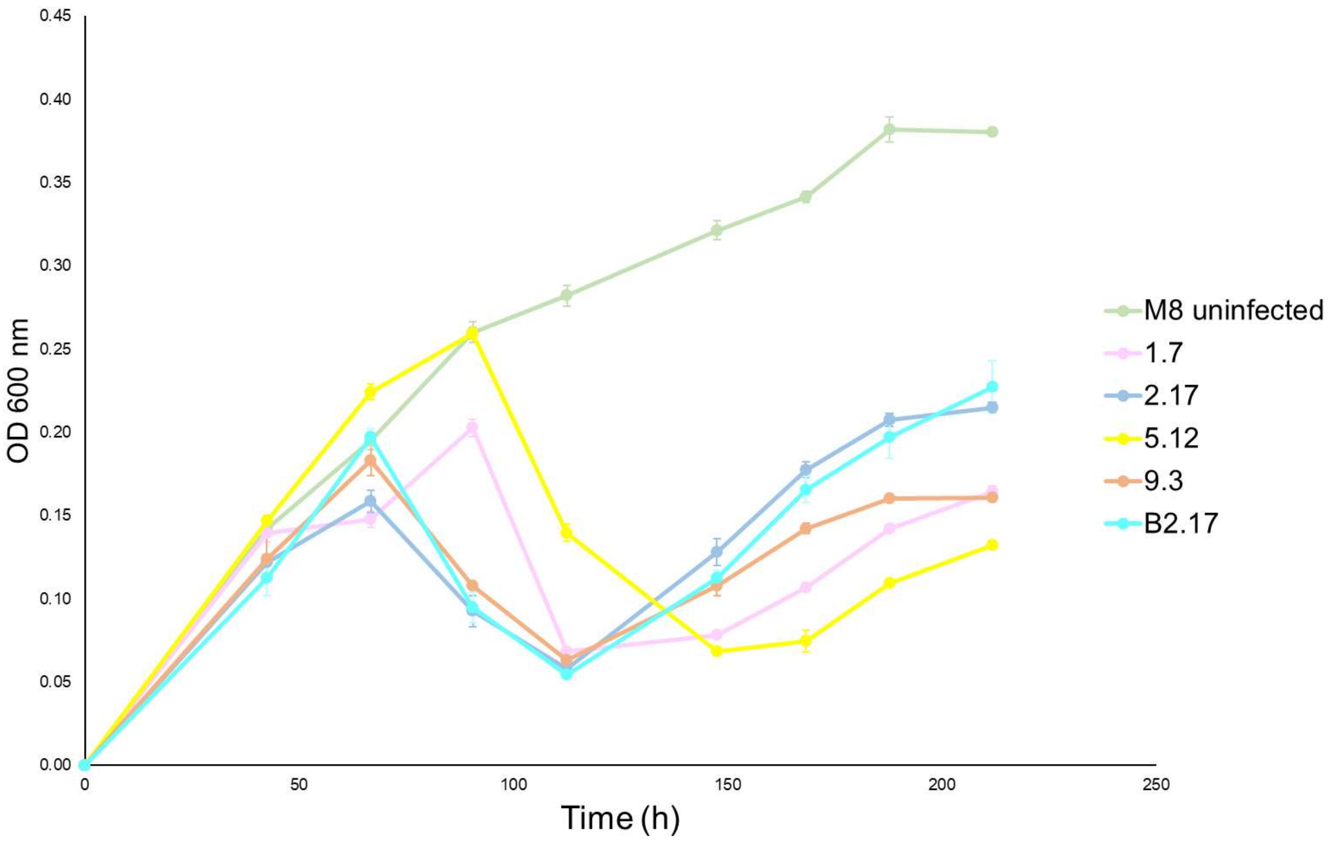

Figure S13: Growth curves of *Sal. ruber* M8 infected with different *Phoenicisaliniviruses*. An uninfected control is included. All the analyses were carried out in triplicate.

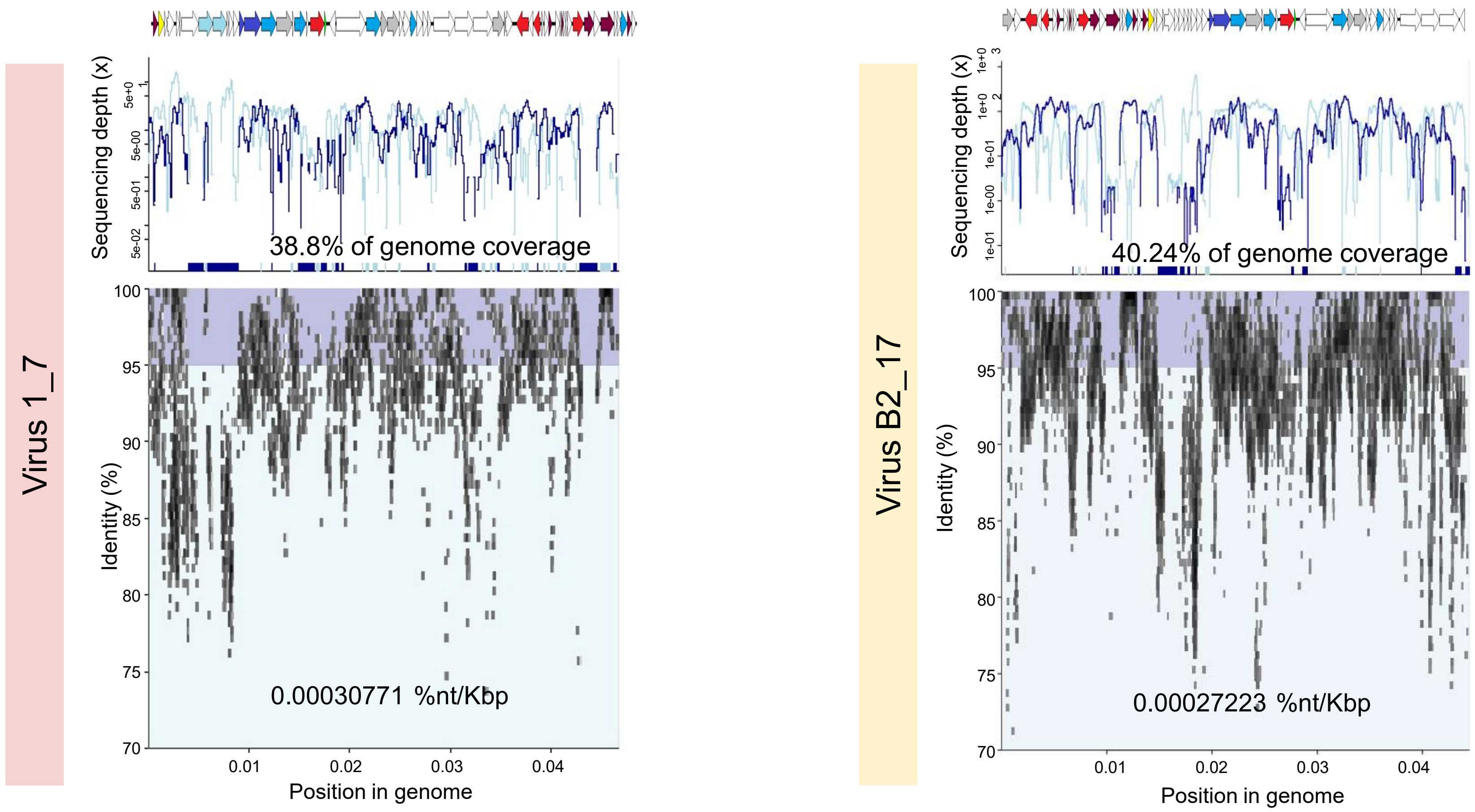

Figure S14. Persistence of *Phoenicisalinivirus* in the system. Recruitment of one genome of each species (left: *P. gimnesicum*; right: *P. balearicum* against viral metagenomic reads from a crystallizer pond from the Es Trenc salterns taken in 2019. Viral genomes are as in Figure 3. The percentage of normalized recruited reads are indicated for each recruitment plot. Relative abundance and genome coverage values were calculated considered only hit over 70% of read coverage and 100% of identity, the values were normalized by metagenome size (total nucleotides) and viral genome length (Kpb).

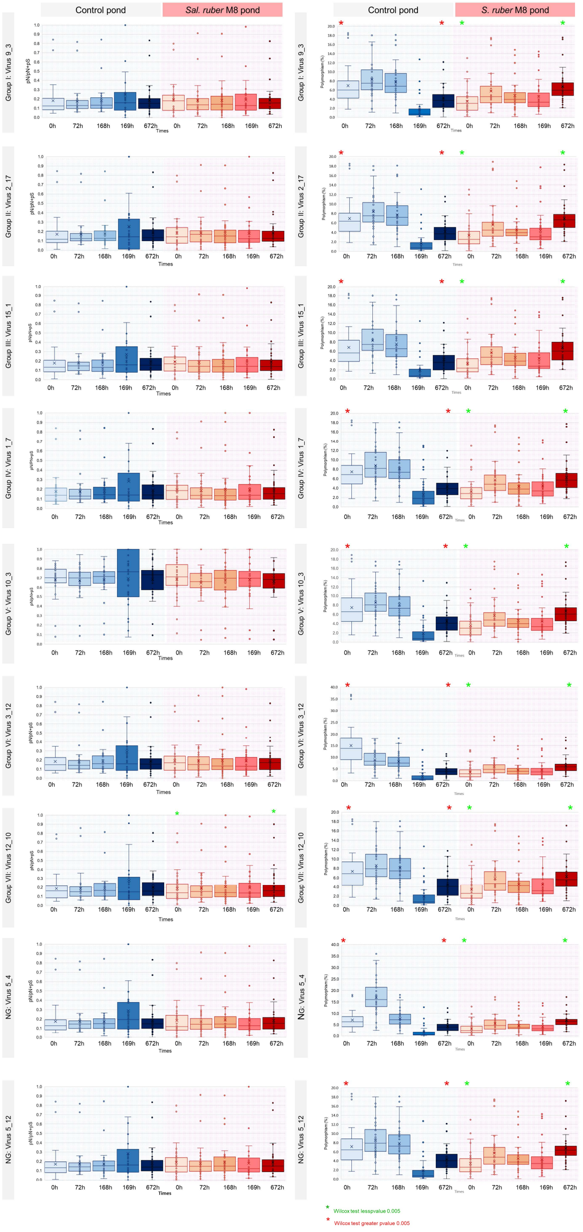

A HIGH QUALITY FIGURE IS ALSO AVAILABLE AS SUPPLEMENTARY MATERIAL

Figure S15. Analyses of SNPs (left) and pN/pN+pS (right) along the experiment of *P. gimnesicum* genomes (one viral genome for each of the 9 groups is shown). Asterisks indicate whether there is a significative (Wilcox test p value <0.005) increase (green) or decrease (red) in the calculated parameters. All changes are against time zero values.

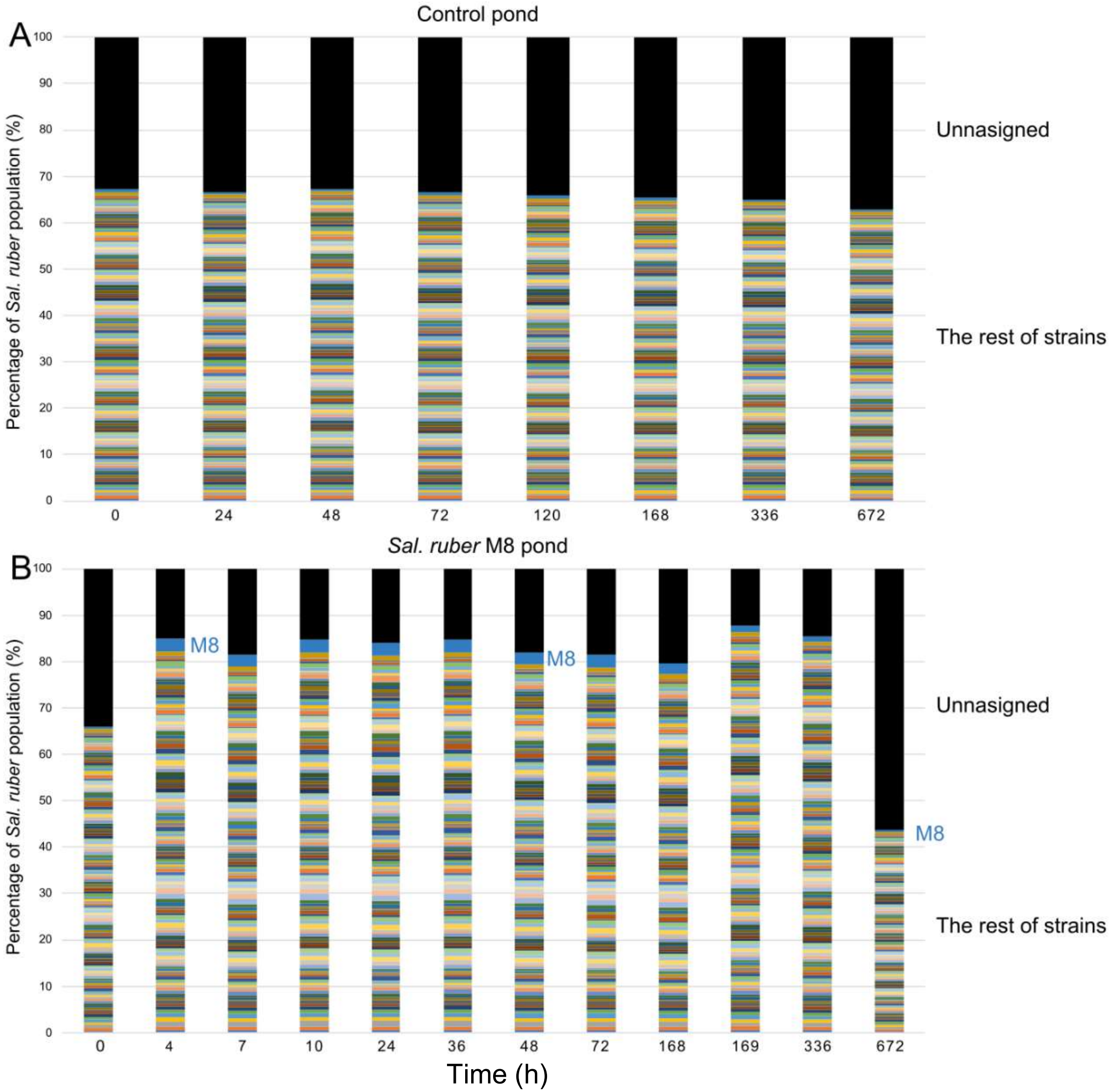

Figure S16. *Sal. ruber* isolates abundance dynamics in (A) the control pond and (B) the pond amended with *Sal. ruber* M8 culture. Metagenomic reads from each time point were aligned against all *Sal. ruber* genomes and the total percentage of *Sal. ruber* population was represented by all metagenomic reads mapping with >95% identity to any genome. The relative abundance of each individual isolate compared to the total *Sal. ruber* population was calculated as the percentage of reads mapping with 100% identity. Black boxes denote the percentage of the total *Sal. ruber* population not represented by the genomes. The source data used is provided in Sup. Dataset S6, second tab. The relative abundance of the strains was calculated by mapping the metagenomic reads to each whole genome sequence individually. Since pairs of genomes may share identical genomic regions, this methodology could overestimate the relative abundance of some strains. However, the analysis is useful for visualizing intraspecific strain dynamics throughout the experiment.
