# Supplementary material for: Experimental evolution at ecological scales allows linking of viral genotypes to specific host strains
Source: ISME J. 2024 Nov 23;18(1):wrae208. doi: 10.1093/ismejo/wrae208 (PMC11631230; doi:10.1093/ismejo/wrae208)
Supplement: Ramos-Barbero_et_al_Supplementary_R_and_M_and_S_Tables_wrae208 [file ramos-barbero_et_al_supplementary_r_and_m_and_s_tables_wrae208.docx]

**SUPPLEMENTARY METHODS**

**Microbial counts and viral morphologies**

Sampled brines were fixed in situ with formaldehyde at 7% final concentration, overnight at 4°C. After fixation, 8.8 ml of PBS (1X) was added, and the mixture was filtered through a 0.22 μm pore filter (Isopore GTTP, Millipore). Filters were stored at 20°C until use. Cell counts were performed by double-staining with 4,6-diamidino-2-phenylindole (DAPI; 1 μg/ml) and fluorescence in situ hybridization (FISH) with EUB338 and Arc915 probes as described by Antón *et al*. (1999). For viral count by flow cytometry, brines were fixed with glutaraldehyde (0.5% final concentration) for 30 minutes at 4°C and stored at -80°C until they were used. The samples were diluted using TE buffer 1X (10 mMTris-HCl, 1 mM EDTA, pH 8) so that the event rate measured was between 300 to 1000 virus-like particles (VLP). Staining was performed using SYBR-gold at a final concentration of 5x10^-5^ commercial stock (Invitrogen catalogue no. S11494, concentration 10,000X), incubated in dark conditions for 10 minutes at 80°C and then the sample cooled before flow cytometer analysis (Brussaard, 2004; Viver *et al.*, 2019). The viral counts were measured using a FACS Canto II cytometer (BD Biosciences) equipped with a 488-nm laser. The data were processed using FACSDiva v.8 Software.

The viral morphologies (from natural samples and isolated viruses) were determined by transmission electron microscopy (TEM), in a Jeol JEM-2010 transmission electron microscope (JEOL Manufacturer, Tokyo, Japan). A total of 5 μl of each viral concentrate (see below in Viral DNA extraction protocol) was stained with 2% uranyl acetate following the protocol previously described in Villamor *et al*., 2018.

**Cell DNA and RNA extraction**

For cell DNA extraction, 25 ml of brine samples were centrifuged at 13,000 rpm and DNA was extracted as detailed by Urdiain (Urdiain *et al.*, 2008). For RNA extraction, pellets were stored in RNAlaterTM solution (Invitrogen, Thermo Fisher Scientific, Lithuania) at -80 ºC until processed. The PowerMax Soil DNA kit for extracting RNA was used for total RNA extractions (MoBio) and TURBO DNAse (Ambion) 2U/µl (enzyme stock 10 U/ml) according to the manufacturer directions up to remove completely the DNA.

**Viral isolation and purification**

An exponentially grown M8 culture was mixed separately with 100 μl of non-diluted, 100-fold and 10^4^-fold diluted filtered viral assemblages sampled from the control or the M8 pond at 9 time points (i.e. 0, 4, 7, 10, 24, 32, 48, 72, and 120 hours). The mixtures were incubated for 30 min at room temperature without shaking. After incubation, the cultures were mixed with 25% SW soft agar, poured into 25% SW+0.2% YE agar plates and incubated at 37 °C for at least 10 days (Villamor *et al.*, 2018). As this assay did not yield any viral plaques a second plaque assay was performed. In this second assay, 5 µL of the viral filtrates were directly spotted on *Sal. ruber* M8 lawns. Plaques were resuspended in SW 25% and reisolated by plaque assay. This is the method described in the main text. All samples taken along the experiment were treated in the same way: first the method described here (direct plating of the viral fraction, obtained after eliminating the cells by filtration) and then, after the lack of results, by spotting the viral fractions on S. ruber M8 lawns and further virus purification (as explained in the main text).

**Chloroform sensitivity**

Chloroform sensitivity of the newly isolated viruses was checked as in Alonso, Rodríguez and Borrego (2002) by mixing 200 µL of phage suspension (10^8^ VLP/ml) with 40 µL of chloroform, shaking vigorously for 1 min and incubating at 30^o^C for 30 min. Then, 10 microlitres of the aqueous phase and of five consecutive decimal serial dilutions were spotted onto *S. ruber* M8 lawns to check for virus activity. An untreated viral suspension was used as control.

**Viral DNA extraction**

To purify the viral fraction, approximately 1 liter of each water sample was filtered by tangential flow filtration (TFF) to remove the cells, and purified viral fractions were concentrated through the Vivaflow 200-PES system (initial volume to 20 ml) with 30 KDa and re-concentrated to 500 μl by ultracentrifugation at 185,000 x g (Beckman Coulter, optima™ Max-XP ultracentrifuge) for 4 h at 20°C. Then, 50 μl of viral ultra-centrifuged sample was used to determine the viral genome sizes by pulsed field gel electrophoresis (PFGE). Viral DNA extraction was carried out as previously described (Santos *et al.*, 2010) incubating low melting agarose inserts (plugs) with Turbo DNase (Ambion) according to the manufacturer indications before ESP treatment, to reduce cellular contamination. The DNA extractions from isolated viruses were carried out separately as detailed above until the β-agarase digestion step. The DNA was then precipitated with a 3 M mixture of sodium acetate pH 5.2 and cold isopropanol (stored at -20°C), with a ratio of 0.1:0.6 volumes. Subsequently, it was kept on ice for 2 minutes and centrifuged for 30 minutes at 13,500 x g at 4°C (Centurion Scientific® K3 series centrifuge, BRK 5424 rotor). Subsequently, 500 μl of 70% ethanol was added and centrifuged for 15 minutes at the same speed and temperature. After centrifugation, the tube was decanted and the DNA pellet was allowed to dry before being resuspended in approximately 15 μl of ultrapure water. The size of each of viral genome was determined by pulsed field gel electrophoresis (PFGE). Viral DNA quality and quantity were measured by QUBIT® 2.0, using the highly sensitive fluorochrome (2–100 ng) kit. Additionally, absence of cell contamination in viral DNA was confirmed by PCR using archaeal and bacterial primers (Gomariz *et al.*, 2014).

**Proteomic analyses**

To extract viral proteins, a total of 10^9^ viruses per sample were separated in a volume of 40 µl to 60 µl, 8 M urea was added (vf: 100 µl) and then, to reduce disulfide bonds, 1mM dithiothreitol (DTT) was added. The mixes were shaken vigorously and incubated 1 hour at room temperature. First, for the cysteine acetylation reaction, 5.5 mM iodoacetamide (IAA) was added and incubated again for 45 minutes in dark conditions. After this time, the urea was diluted to a concentration of 2 M, adding 1% trifluoroacetic acid (TFA). Subsequently, the LysC enzyme mixture (Saveliev *et al.*, 2013; Tsiatsiani *et al.*, 2015) and Trypsin were added to a concentration of 0.225 µg/µl, and incubated overnight at room temperature. Lastly, TFA was added at 1% final concentration to prepare the sample for the subsequent step. The next stage is called “stage tip”, which consists of passing the peptide digestion through a silica membrane (Octadecyl C18 bonded silica) inserted into a 200 µl pipette tip (Rappsilber *et al.*, 2007). In this way, the peptides are retained on this membrane and can be washed and later recovered in solution. Next, the membrane inserted into the tips was washed by adding 50 µl of 100% methanol and centrifuged for 2 minutes at 657 x g in an Eppendorf Model 5810 centrifuge with an Eppendorf® A-2 DWP rotor. This centrifuge was used throughout the process. Subsequently, 50 µl of 100% acetonitrile was added and again removed by centrifugation (2 min, 657 x g). Finally, 50 µl of 0.2% acetonitrile with 0.1% TFA were added, which was removed under the same conditions as in the previous step. Once the membrane was washed, a maximum of 200 µl of the digestion was added each time in order to pass the entire volume of the digestion through the membrane; it was then centrifuged for 10 minutes at 657 x g. Once the peptides were retained on the membrane, they were washed with 50 µl of 2% acetonitrile and 0.1% TFA. The eluate was discarded, and the membrane was transferred to a new collection tube for subsequent elution of the peptides with 80% acetonitrile. The peptides in solution were concentrated in the SpeedVac for 10 minutes to a final volume of 2 µl. Finally, they were resuspended in 40 µl of 2% acetonitrile and 0.1% TFA and stored at -20°C until sequencing by orbital mass spectrometry (Rappsilber *et al.*, 2007). The spectrophotometer model used was the Orbitrap Fusion with UltiMate 3000 RSL Cano System (Thermo Scientific®), especially indicated for difficult samples and those with high salt concentrations.

The proteomes were compared against custom databases (isolate viral genomes from this work) using Maxquant v 2.5.0.0 (Tyanova *et al.*, 2016) in order to identified viral structural protein. Proteomic custom databases included non-redundant ORFs from isolated viral genomes. Only proteins identified with at least two peptides and intensity greater than 1% were considered in the analyses.

**Viral genome diversity analyses**

As recommended by Coutinho *et al*. (2019) to estimate the percentage of polymorphic sites and pN/pS ratios (Schloissnig *et al.*, 2013; Coutinho *et al.*, 2019), only the reads with coverage equal or higher than 5 and codon mutations detected at least four times in at least 1% of mapped reads, were considered. Regarding the diversity data, we have included in the study the values of pN and pS=0, and we have avoided the ∞ (indetermination values) using the approximation pN/pN+pS. This estimation is similar to the ratio dN/dS described by Shloissing in 2013

SNPs and pN/pN+pS ratios were compared between pairs of samples using Wilcoxon rank-sum tests implemented in the coin package (Hothorn *et al.*, 2006) of RStudio v 1.41106 (http:// www.rstudio.com). First, the significant differences (p-value equal or >0.05) between samples were tested using the ‘two side’ option, which was then followed by the ‘less and greater’ option to ascertain the direction of the change.

**qPCR and metabarcoding analyses**

Primers were designed to target the tail fiber protein gene (primers C; 262F_C: TGGGACCAGAACGGGAATCT; 411R_C: TGCCCAAGATGTCGGATCTG), which was highly conserved between genomes, and the capsid protein gene, which was highly variable between genomes (primers RV; 71F_RV: CGCCTGATCTGTCCGACTAC; 220R_RV: GGTAGGACTGAAGCTCGTCG) using Geneious 6.1. Specificity was confirmed in vitro using *Sal. ruber* and DNA extracted from a crystallizer pond from Alicante salterns (CR30). For qPCR analyses, PowerSYBR Green PCR Master Mix (Applied Biosystems) was employed with 0.2 nM of primers and the following PCR program in a StepOnePlus Real-Time thermocycler (Applied Biosystems): 95°C for 10 minutes, 50 cycles of 95°C 15 seconds and 60°C 1 minute and a final melting step raising temperature from 60°C to 95°C and reading fluorescence each 0.3°C. To obtain absolute quantifications, viral DNA obtained from cultures and quantified by Qubit 2.0 (Life Technologies) was used as standard. Six 1:10 dilutions were employed to calculate the standard curve. All qPCR controls and samples were carried out in triplicate. For metabarcoding analyses, Illumina adapter sequences were added to the primers mentioned above and the cellular DNA from control and *Sal. ruber* pond from times 0h and 672h was amplified by PCR using the Taq DNA polymerase, recombinant (Invitrogen) in a SimpliAmp thermal cycler (Applied Biosystems) with the following PCR program: 94°C for 3 minutes, 30 cycles of 94°C 30 seconds, 60°C 1 minute and 72°C 2 minutes followed by a final extension step of 72°C 30 minutes. Amplicons were sequenced at FISABIO (Fundación para el Fomento de la Investigación Sanitaria y Biomédica, Valencia, Spain) using an Illumina Nextera 2 x 250 bp, paired-end reads run.

**Growth curves of infected *Sal. ruber* M8**

Three ml of an exponentially growing culture of *Sal. ruber* (OD600= 0.3) were mixed with 300 µL of a viral suspension (10^8-^10^9^ VLPs/ml, depending on the virus) and left for 30 min at room temperature. Then, 72 ml of culture medium (SW25%, YE 0.2%) were added and the culture split into three falcon tubes which were incubated at 37oC (30 rpm, tilted at 30 degrees approx.). Growth was monitored by OD600 reading every 24 hours approx. An uninfected culture was included as a control.

**SUPPLEMENTARY RESULTS AND DISCUSSION**

**Isolation of viruses infecting *Sal. ruber* M8**

Around 40% of the 119 viral genomes belonged to Group I, that included viruses from the three sources (pond M8 at times 360 and 672 h, and pond 2 at 672 h; see Table S2), whereas the rest of groups contained viruses isolated from only one of the sources. Viruses isolated from the final time point encompassed a wider genomic diversity since they belonged to 6 out of the 9 groups.

The isolation of viruses infecting *Sal. ruber* M8 from a contiguous pond amended with another *Salinibacter* strain may be due to the presence of these viruses, albeit in very low concentrations, before the experiment, as in the control pond. This is related with the well-known fact that culture can retrieve members of the rare biosphere (Pedrós-Alió, 2012) as well as, occasionally, abundant microbes. However, the relevant point addressed here is the dynamics of the viral natural populations in the amended pond which is followed using the isolated viral genomes as references.

**Phoenicisalinivirus genome annotation**

Some of the annotated genes corresponded to proteins typical of the head and tail morphology observed under TEM, such as terminases and capsid structural proteins. In addition, *Phoenicisalinivirus* virions likely carry a peptidoglycan degrading enzyme since the N-acetylmuramoyl-L-alanine amidase gene product was among the proteins detected by mass spectrometry. These lysin-encoding genes are frequently present in bacteriophages and are involved in the initial degradation of the cell wall needed for viral infection (Vaquez and Briers, 2023).

All *Phoenicisalinivirus* genomes had genes coding for integrases and could thus be temperate viruses. In agreement with this, *att* regions could also be detected in their genomes. These regions had high identities with homologous regions in the genome of strain *Sal. ruber* M31, which was isolated in 1999 from these salterns. As with the previously described Kryptosaliniviruses (Villamor *et al.*, 2018) , phoenicisaliniviruses seem to be purely lytic for their host (*Sal. ruber* M8) under the assayed conditions, which raises the question as to whether there are alternative hosts/conditions in which these viruses enter the lysogenic cycle. In addition, polymerases could not be identified in the *Phoenicisalinivirus* genomes contrary to what was found in the previously isolated saliniviruses (Villamor *et al.*, 2018), whereas genes coding for tRNAs were found only in the *P. balearicum* representative. A gene coding for a nuclease with a high identity (70% aa) with that of *Salinibacter* strains as well as one environmental halophage (Garcia-Heredia *et al.*, 2012) was also detected. This finding supports the use of restriction enzyme genes in viral genomes as a way of assigning viruses to their hosts.

The most conspicuous trait in the *Phoenicisalinivirus* genomes was the presence of a gene coding for a ribosomal protein, with a 100% identity at the amino acid level with an S15 protein encoded in a prophage integrated into *Sal. ruber* strain SP2008, which was isolated in 2016 from a coastal solar saltern in mainland Spain. However, the identity with *Sal. ruber* M8 ribosomal protein S15 was only 46%, and the viral protein was 14 amino acids shorter. Thus, it was clearly different from the *Sal. ruber* S15 protein. Ribosomal proteins have recently been found in viral genomes(Mizuno *et al.*, 2019) and metagenomes, although S15 had been found only in human and animal associated viral metagenomes.

**Mutation analyses**

In order to monitor the evolution of whole viral genomes, and not only the fraction corresponding to the above discussed islands, SNPs and the ratio of non-synonymous to synonymous mutations (Figure S15 and Supplementary Dataset S4) in the metagenomes were analyzed using the viral genomes as reference. These analyses only included the genes that could be detected over a given threshold (see the Methods section) at each analyzed point (pond + time) and that some genes may be shared in the natural community with viral genomes other than the species analyzed here (as shown by the recruitment plots). The number of SNPs in the *Phoenicisalinivirus* genomes increased in the M8 pond and decreased in the control pond (Figure S9), likely due to the population size increase experienced by the virus in the M8 pond, as observed for marine viruses (Ignacio-Espinoza *et al*., 2023). Regarding pN/pN+pS analyses, no significant changes along the experiment were found for whole genomes within *P. gymnesicum* (Figure S15).

**Dynamics of (culturable) *Sal. ruber* strains along the experiment**

In order to get a deeper insight on the composition of the *Sal. ruber* assemblage along the experiment, the genomes of 131 *Sal. ruber* strains isolated from the same system were recruited against the metagenomes of control and M8 ponds (Supplementary Figure S16 and Supplementary Dataset S6). These isolated strains accounted for 67% of the reads constituting the *Sal. ruber* population at the beginning of the experiment in both ponds. However, at the end of the experiment, this fraction decreased to 43% in the amended pond (and only to 62% in the control) indicating that other strains, not represented in the 131 strain pool, increased their abundance along the experiment.

**References for Supplementary Results and Methods not cited in the main text**

Alonso, M.C., Rodríguez, J., and Borrego, J.J. (2002) Characterization of marine bacteriophages isolated from the Alboran Sea (Western Mediterranean). *J Plankton Res* **24**: 1079–1087.

Brussaard, C.P.D. (2004) Optimization of procedures for counting viruses by flow cytometry. *Appl Environ Microbiol* **70**: 1506–1513.

Cantu, V.A., Sadural, J., and Edwards, R. (2019) PRINSEQ++, a multi-threaded tool for fast 1 and efficient quality control and 2 preprocessing of sequencing datasets. *PeerJ Preprints* **7**: e27553v1

Coutinho, F.H., Rosselli, R., and Rodríguez-Valera, F. (2019) Trends of Microdiversity Reveal Depth-Dependent Evolutionary Strategies of Viruses in the Mediterranean. *mSystems* **4**: e00554-19.

Gomariz, M., Martínez-García, M., Santos, F., Rodriguez, F., Capella-Gutiérrez, S., Gabaldó, T., *et al*. (2014) From community approaches to single-cell genomics: the discovery of ubiquitous hyperhalophilic Bacteroidetes generalists. *ISME J* **9**: 16–31.

Hothorn, T., Hornik, K., Van De Wiel, M.A., and Zeileis, A. (2006) A lego system for conditional inference. *Am Stat* **60**: 257–263.

Langmead, B. and Salzberg, S.L. (2012) Fast gapped-read alignment with Bowtie 2. *Nat Methods* **9**: 357–359.

Mizuno, C.M., Guyomar, C., Roux, S., Lavigne, R., Rodriguez-Valera, F., Sullivan, M.B., *et al*. (2019) Numerous cultivated and uncultivated viruses encode ribosomal proteins. *Nat Commun* **10**: 752.

Pedrós-Alió C. (2012) The Rare Bacterial Biosphere. *Ann Rev Mar Sci* **4**: 449–466.

Rappsilber, J., Mann, M., and Ishihama, Y. (2007) Protocol for micro-purification, enrichment, pre-fractionation and storage of peptides for proteomics using StageTips. *Nat Protoc* **2**: 1896–1906.

Saveliev, S., Bratz, M., Zubarev, R., Szapacs, M., Budamgunta, H., and Urh, M. (2013) Trypsin/Lys-C protease mix for enhanced protein mass spectrometry analysis. *Nat Methods* **10**: i–ii.

Schloissnig, S., Arumugam, M., Sunagawa, S., Mitreva, M., Tap, J., Zhu, A., *et al*. (2013) Genomic variation landscape of the human gut microbiome. *Nature* **493**: 45–50.

Trapnell, C., Pachter, L., and Salzberg, S.L. (2009) TopHat: Discovering splice junctions with RNA-Seq. *Bioinformatics* **25**: 1105–1111.

Tsiatsiani, L., Heck, Albert J R, Heck, A J R, and Bijvoet, P. (2015) Proteomics beyond trypsin. *FEBS J* **282**: 2612–2626.

Tyanova, S., Temu, T., and Cox, J. (2016) The MaxQuant computational platform for mass spectrometry-based shotgun proteomics. *Nat Protoc* **11**: 2301–2319.

Urdiain, M., López-López, A., Gonzalo, C., Busse, H.J., Langer, S., Kämpfer, P., and Rosselló-Móra, R. (2008) Reclassification of Rhodobium marinum and Rhodobium pfennigii as Afifella marina gen. nov. comb. nov. and Afifella pfennigii comb. nov., a new genus of photoheterotrophic Alphaproteobacteria and emended descriptions of Rhodobium, Rhodobium orientis and Rhodobium gokarnense. *Syst Appl Microbiol* **31**: 339–351.

Vaquez, R. and and Briers, Y. (2023) What ’ s in a Name ? An Overview of the Proliferating Nomenclature in the Field of Phage Lysins. *Cells* **12**: 2016.

Weeks, N.T. and Luecke, G.R. (2017) Optimization of SAMtools sorting using OpenMP tasks. *Cluster Comput* **20**: 1869–1880.

**Table S1.** Microbial counts (DAPI, FISH and SYBR-gold) and physico-chemical characteristics of the hypersaline water over the experiment.

| **Pond** | **Time (hours)** | **Salinity (%)** | **pH** | **T (°C)** | **Cells/mL** | **Ste. dev Cells/mL** | **Archaea/mL** | **Bacteria/mL** | **VLP/ml** | **Ste dev. VLP/ml** | **ΔVLP** | **ΔCells** |
| --- | --- | --- | --- | --- | --- | --- | --- | --- | --- | --- | --- | --- |
| **Control** | 0 | 32.8 | 7.17 | 39 | 5.5X10^7^ | 2.5X10^5^ | 4.4X10^7^ | 1.5X10^7^ | 6.96X10^7^ | 3.3X10^7^ | 1.00 | 1.00 |
|  | 4 | 34 | 7.14 | 39 | 5.0X10^7^ | 9.9X10^6^ | 3.6X10^7^ | 1.5X10^7^ | - | - | - | 0.90 |
|  | 24 | 32 | 7.15 | 39 | 5.5X10^7^ | 2.5X10^7^ | 3.6X10^7^ | 1.9X10^7^ | 7.50 X10^7^ | 3.2X10^7^ | 1.08 | 1.00 |
|  | 48 | 32.4 | 7.19 | 32 | 5.7X10^7^ | 5.7X10^7^ | 4.1X10^7^ | 1.6X10^7^ | 4.82 X10^7^ | 1.4X10^7^ | 0.69 | 1.00 |
|  | 72 | 34 | 7.12 | 33 | 5.1X10^7^ | 1.3X10^5^ | 3.5X10^7^ | 1.5X10^7^ | 4.08 X10^7^ | 3.0X10^7^ | 0.59 | 0.93 |
|  | 168 | 32.8 | 7.1 | 33 | 5.4X10^7^ | 1.2X10^6^ | 3.9X10^7^ | 1.7X10^7^ | 8.65 X10^7^ | 2.6X10^7^ | 1.24 | 0.98 |
|  | 169** | 25.6 | 7.71 | - | 6.4X10^7^ | 8.1X10^6^ | 2.2X10^7^ | 3.9X10^7^ | 8.41 X10^7^ | 4.8 X10^7^ | 1.21 | 1.15 |
|  | 336 | 31 | 7.36 | 27 | 5.3X10^7^ | 5.9X10^6^ | 3.1X10^7^ | 1.6X10^7^ | 9.53 X10^7^ | 3.2 X10^7^ | 1.37 | 0.96 |
|  | 672 | 34 | 7.36 | 27 | 4.9X10^7^ | 1.4X10^6^ | 3.6X10^7^ | 1.2X10^7^ | 6.57 X10^7^ | 0 | 0.94 | 0.88 |
| ***Sal. ruber***  **M8** | 0 | 32.4 | 7.4 | 36 | 4.8X10^7^ | 7.5X10^6^ | 3.5X10^7^ | 1.2X10^7^ | 6.1X10^6^ | 3.9X10^6^ | 1.00 | 1.00 |
|  | 4* | 32.4 | 7.28 | 36 | 6.6 X10^7^ | 6.1X10^6^ | 2.4X10^7^ | 4.1X10^7^ | - | - | - | 1.37 |
|  | 24 | 31.5 | 7.32 | 35 | 4.4 X10^7^ | 4.4X10^6^ | 2.3X10^7^ | 2.0X10^7^ | 7.5X10^6^ | 6.2X10^6^ | 1.23 | 0.91 |
|  | 48 | 33 | 7.27 | 29 | 5.7 X10^7^ | 5.7X10^7^ | 2.8X10^7^ | 2.7X10^7^ | 6X10^6^ | 4.6X10^6^ | 1.00 | 1.17 |
|  | 72 | 33.6 | 7.3 | 35 | 5.4 X10^7^ | 5.9X10^5^ | 2.8X10^7^ | 2.5X10^7^ | 6.2X10^6^ | 4.9X10^6^ | 1.02 | 1.10 |
|  | 168 | 31.8 | 7.57 | 31 | 5.4 X10^7^ | 1.2X10^6^ | 3.9X10^7^ | 2.1X10^7^ | 1.3X10^7^ | 8.9X10^6^ | 2.24 | 1.12 |
|  | 169** | 27.2 | 7.36 | - | 5.8 X10^7^ | 6.5X10^6^ | 2.5X10^7^ | 3.1X10^7^ | 2.2X10^7^ | 5.1X10^6^ | 3.61 | 1.2 |
|  | 336 | 32.8 | 7.22 | 27 | 4.7 X10^7^ | 8.2X10^6^ | 3.2X10^7^ | 1.5X10^7^ | 1.8X10^7^ | 6.1X10^6^ | 2.99 | 0.97 |
|  | 672 | 34.4 | 7.4 | 30 | 5.1 X10^7^ | 5.3X10^6^ | 4.0X10^7^ | 1.1X10^7^ | 3.3X10^7^ | 2.5X10^6^ | 5.52 | 1.00 |

(-) No data. **Salinibacte*r *ruber* M8. inoculation. **Refilling point.

**Table S2.** Phoenicisaliniviruses isolates selected for this study

|  | **Group** | **Pond** | **Time** | **Isolated viruses** |
| --- | --- | --- | --- | --- |
| *Phoenicisalinivirus gymnesicum* | 1 | M8 | 360 | 11_1, 11_2, 11_3, 11_4, 11_5, 11_6, 11_7, 11_8 |
|  |  |  | 672 | 1_1, 1_2, 1_3, 1_4, 1_5, 1_6, 1_8, 1_10, 1_11, 1_12, 1_13, 1_14, 1_15, 1_16, 1_17, 1_18, 1_19, 1_20, 1_21, 1_22, 1_24, 1_25, 1_26, 1_27, 2_10, 2_11, 5_1, 5_2, 5_3, 5_5, 5_10, 5_15, 7_1, 7_2, 7_3, 7_5, 7_6, 7_7, 7_8, 7_9, 7_10, 7_11, 7_12 |
|  |  | E2^a^ | 672 | 8_2, 8_3, 8_4, 8_5, 8_6, 8_7, 8_8, 8_9, 8_10, 8_11, 8_12, 8_13, 8_15, 8_16, 8_17, 9_1, 9_2, 9_3* |
|  | 2 | M8 | 672 | 2_1, 2_2, 2_3, 2_4, 2_5, 2_6, 2_7, 2_8, 2_9, 2_12, 2_13, 2_14, 2_15, 2_16, 2_17*, 4_1, 4_2, 4_3, 4_4, 4_5, 4_6, 4_7, 4_8, 4_9, 4_10, 4_11, 4_12, 4_13, 4_14, 4_15, 4_16, 4_17, 4_18, 4_19, 4_20, 4_21, 4_22 |
|  | 3 | M8 | 360 | 15_1*, 15_2, 15_3, 15_4, 15_5 |
|  | 4 | M8 | 360 | 14_1, 14_2, 14_3, 14_4, 14_5, 14_6, 14_7 |
|  |  |  | 672 | 1_7*, 1_9 |
|  | 5 | E2** | 672 | 10_2, 10_3*, 10_4, 10_5, 10_6 |
|  | 6 | M8 | 672 | 3_1, 3_2, 3_3, 3_4, 3_5, 3_6, 3_7, 3_8, 3_9, 3_10, 3_11, 3_12*, 3_13, 3_14, 3_15, 3_16, 3_17, 3_18, 3_19, 3_20, 5_9, 5_14, 6_2, 6_3, 6_5, 6_8, 6_10, 6_12, 6_13, 6_14, 6_15, 6_16, 6_17 |
|  | 7 | M8 | 360 | 12_1, 12_2, 12_3, 12_4, 12_5, 12_6, 12_7, 12_8, 12_9, 12_10*, 12_11, 13_1, 13_2, 13_3, 13_4, 13_5, 13_6, 13_7, 13_8, 13_9 |
|  | NG^b^ | M8 | 672 | 5_4*, 5_12* |
|  | PB^c^ | M8 | 672 | B2_17* |

*Virus selected as representatives of their groups. E2^a^: A contiguous pond amended with another *Salinibacter* species, not included in the experiment. NG^b^: virus not belonging to any of the previous groups, according to the genome similarity based analysis. PB^c^: Single isolated representative of the species *Phoenicisalinivirus balearicum*
