# Supplementary figures and images for: Experimental evolution at ecological scales allows linking of viral genotypes to specific host strains
Source: ISME J. 2024 Nov 23;18(1):wrae208. doi: 10.1093/ismejo/wrae208 (PMC11631230; doi:10.1093/ismejo/wrae208)

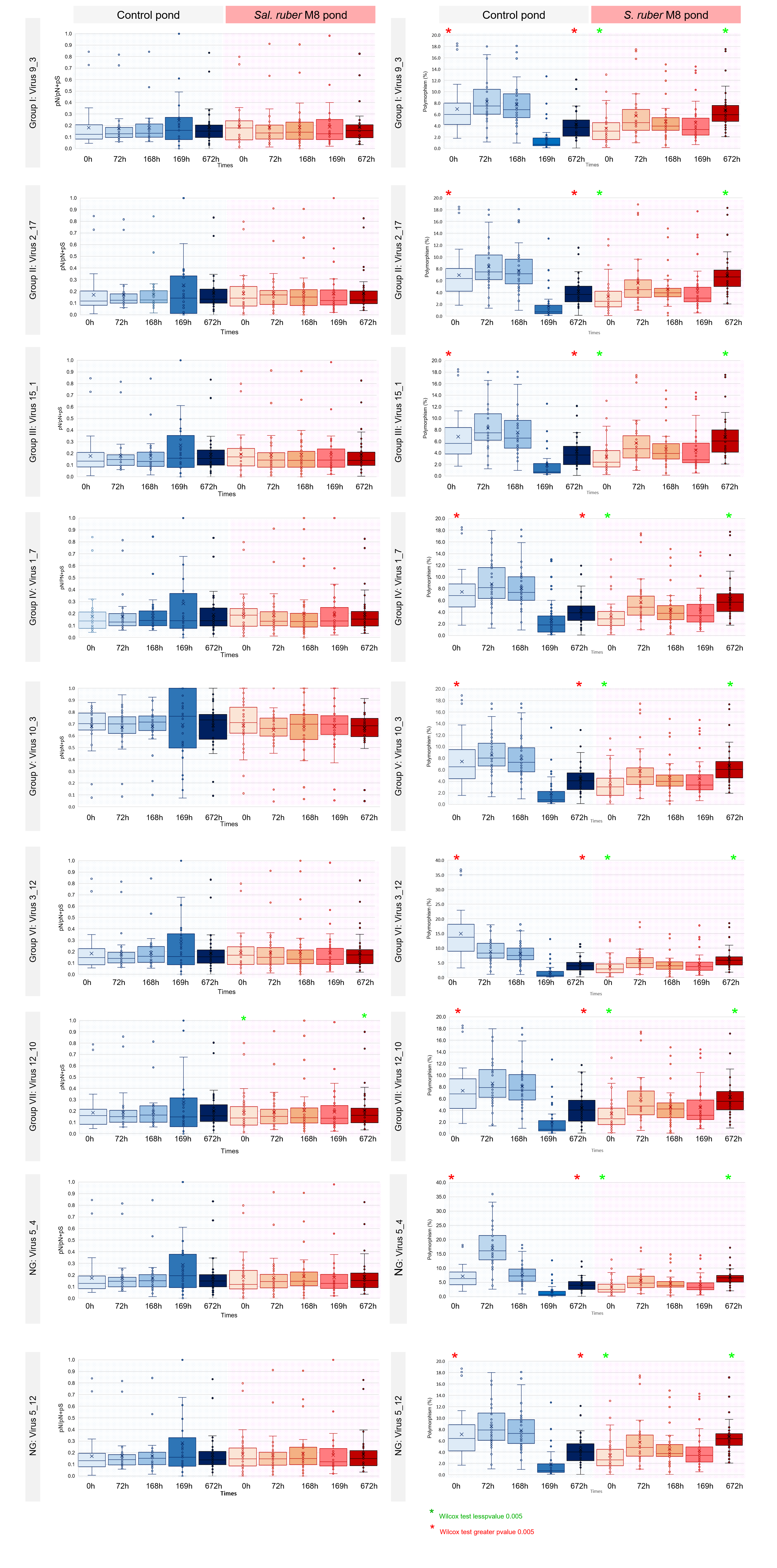

Supplement: FigureS15_wrae208 [file figures15_wrae208.jpeg]
